# Meta-analytic evidence for the anti-aging effect of hormesis on *Caenorhabditis* *elegans*

**DOI:** 10.18632/aging.102773

**Published:** 2020-02-07

**Authors:** Tao Sun, Huifeng Wu, Ming Cong, Junfei Zhan, Fei Li

**Affiliations:** 1 CAS Key Laboratory of Coastal Environmental Processes and Ecological Remediation, Yantai Institute of Coastal Zone Research (YIC), Chinese Academy of Sciences (CAS); Shandong Key Laboratory of Coastal Environmental Processes, YICCAS, Yantai 264003, P. R. China; 2 Laboratory for Marine Fisheries Science and Food Production Processes, Qingdao National Laboratory for Marine Science and Technology, Qingdao 266237, P. R. China; 3 University of Chinese Academy of Sciences, Beijing 100049, P. R. China; 4 Center for Ocean Mega-Science, Chinese Academy of Sciences, Qingdao 266071, P. R. China

**Keywords:** hormesis, anti-aging, aging, *Caenorhabditis elegans*, meta-analysis

## Abstract

Mild stress-induced hormesis, as a promising strategy to improve longevity and healthy aging, meets both praise and criticism. To comprehensively assess the applicability of hormesis in aging intervention, this meta-analysis was conducted focusing on the effect of hormesis on *Caenorhabditis elegans*. Twenty-six papers involving 198 effect size estimates met the inclusion criteria. Meta-analytic results indicated that hormesis could significantly extend the mean lifespan of *C. elegans* by 16.7% and 25.1% under normal and stress culture conditions (*p* < 0.05), respectively. The healthspan assays showed that hormesis remarkably enhanced the bending frequency and pumping rate of worms by 28.9% and 7.0% (*p* < 0.05), respectively, while effectively reduced the lipofuscin level by 15.9% (*p* < 0.05). The obviously increased expression of *dauer formation protein-16* (1.66-fold) and its transcriptional targets, including *superoxide dismutase-3* (2.46-fold), *catalase-1* (2.32-fold) and *small heat shock protein-16.2* (2.88-fold) (*p* < 0.05), was one of the molecular mechanisms underlying these positive effects of hormesis. This meta-analysis provided strong evidence for the anti-aging role of hormesis, highlighting its lifespan-prolonging, healthspan-enhancing and resistance-increasing effects on *C. elegans*. Given that *dauer formation protein-16* was highly conservative, hormesis offered the theoretical possibility of delaying intrinsic aging through exogenous intervention among humans.

## INTRODUCTION

Hormesis, a biphasic dose-response characterized by low-dose stimulation and high-dose inhibition, is now considered as a significant toxicological concept to account for the beneficial effects of mild stress [1, 2]. The concept of hormesis arouses great interest, because it is a near-universal and reproducible phenomenon with profound implications for the clinical trial, drug discovery and risk assessment [3]. As a beneficial compensatory response triggered by mild stress, hormetic individuals generally exhibit better performance than the untreated controls, and the potential anti-aging effect of hormesis has attracted more attention [4, 5]. It seems promising to apply hormesis in aging intervention, which is evidenced by multiple studies, like the beneficial effects of moderate exercise-induced hormesis on body function and aging-related diseases [6]. However, there are still considerable debates regarding the origin and mechanisms of aging and hormesis, such as the conflicting evidence related to the role of ROS in aging [7, 8], and the hormetic effect manifested by stress-induced cost-free benefits or trade-offers with other fitness traits [9, 10]. More importantly, the application of hormesis in aging therapy and interventions is in its infancy, and there are limited investigations in this field [11]. Therefore, the anti-aging effect of hormesis remains controversial [4].

Some researchers indicated the anti-aging effect of hormesis, and considered it as an overcompensation response to the disruption in homeostasis [12]. According to the investigations, hormesis could not only contribute to homeostasis re-establishment, but also strengthen the defense system and immune function by inducing expression of molecular chaperones and immunologic factors with potential anti-aging effect [13, 14]. However, others argued that hormesis might accelerate aging rather than delay it. They proposed that the appearance of hormesis was often accompanied by mild or severe cell damages [15]. Because hormesis could significantly promote the rate of cell proliferation, the occurrence of damages could not be recognized and repaired by the immune system timely and exactly, thereby resulting in damage accumulation and aging acceleration [16, 17]. There is an antinomy between hormesis and anti-aging: hormesis is considered to delay aging, but also accelerate it [18]. Hence, further explorations are needed to determine whether hormesis can delay aging.

At present, most researchers take a wait-and-see attitude to hormetic treatment for human health, due to the contradictory evidence. It is meaningful to conduct a systematic assessment on the existed evidences in the absence of large-scale empirical research on the correlation between hormesis and aging/anti-aging. Meta-analysis is a powerful tool to synthesize multiple or even conflicting evidence to get a clear and reliable final-evidence, achieving the purpose of quantitative review [19]. The application of meta-analytic method contributed a lot to clarifying whether a treatment had anti-aging effect. For example, Liu et al [20] combined 18 studies regarding the effect of growth hormone supplementation on aging, indicating that growth hormone was not only an invalid anti-aging therapy, but also associated with high rates of adverse events. Peterson et al [21] pooled 47 studies about the relationship between resistance exercise and muscular strength, identifying their positive correlation, thereby resistance exercise was a viable strategy to prevent aging-related muscular weakness. On these grounds, in order to thoroughly assess the effect of hormesis on aging, in this work, 26 papers documenting the changes of aging-related indicators induced by hormesis in *Caenorhabditis elegans* (a significant model organism in aging research due to its unique biological features like short life cycle, strong reproductive ability and clear genetic background [22]) were meta-analyzed. This study highlighted the positive effects of hormesis on lifespan, healthspan and stress resistance in *C. elegans*. These findings may be discrepant with human clinical experiments. However, it is predictable that low-level stress treatment, as a potential aging intervention strategy, has a bright future.

## RESULTS

### Overview of included studies

**Figure 1:**
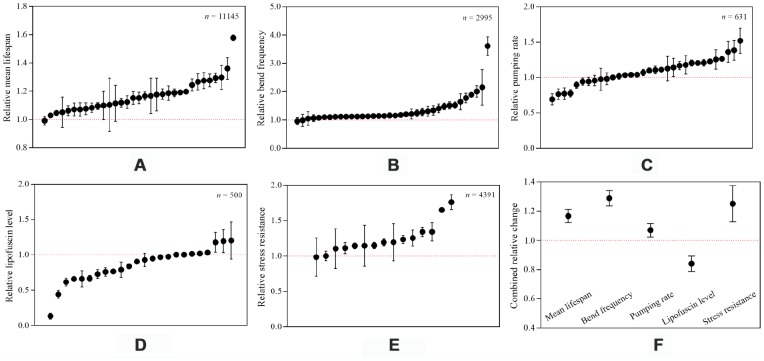
**Relative changes of involved indicators.** (**A**–**E**) denoted the relative changes of mean lifespan, bend frequency, pumping rate, lipofuscin level and stress resistance of *Caenorhabditis elegans* in hormesis groups compared to control groups, while (**F**) represented the combined relative change of each indicator based on random-effect model. The number of worms included in each indicator was shown in its corresponding graph. Data were presented by mean with 95% CIs.

**Figure 2:**
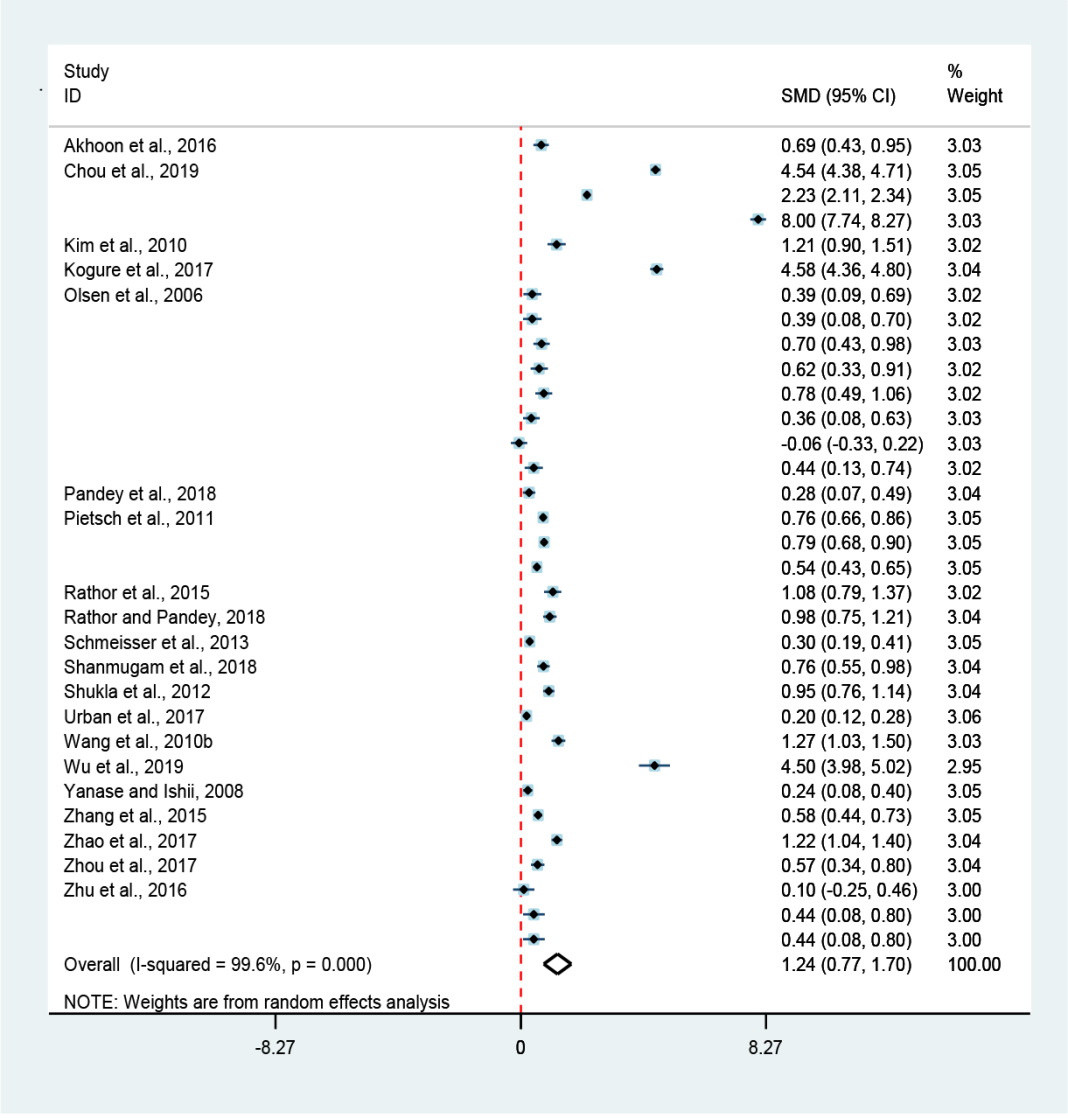
**Association of hormesis and mean lifespan.** The diamond represented the combined effect size of mean lifespan. The red-dotted-line was invalid, and if the diamond did not intersect with it, meaning that hormesis could significantly extend (on the right) or limit (on the left) the mean lifespan of *C. elegans* (*p* < 0.05).

**Figure 3:**
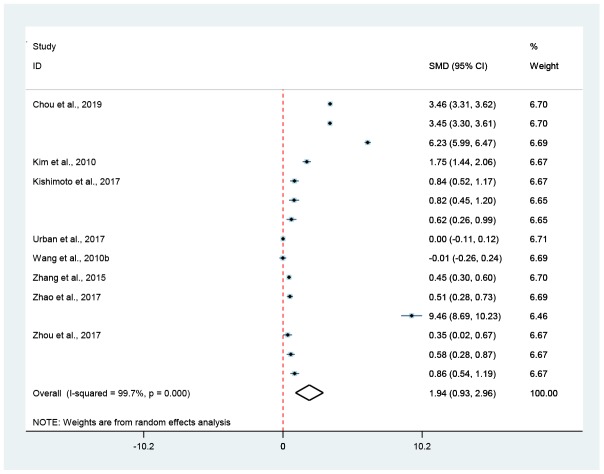
**Association of hormesis and bend frequency.** The pooled effect size of bend frequency was shown by the diamond. The diamond did not intersect with the red-dotted-line, indicating that hormesis could remarkably improve (on the right) or reduce (on the left) the bend frequency of *C. elegans* (*p* < 0.05).

**Figure 4:**
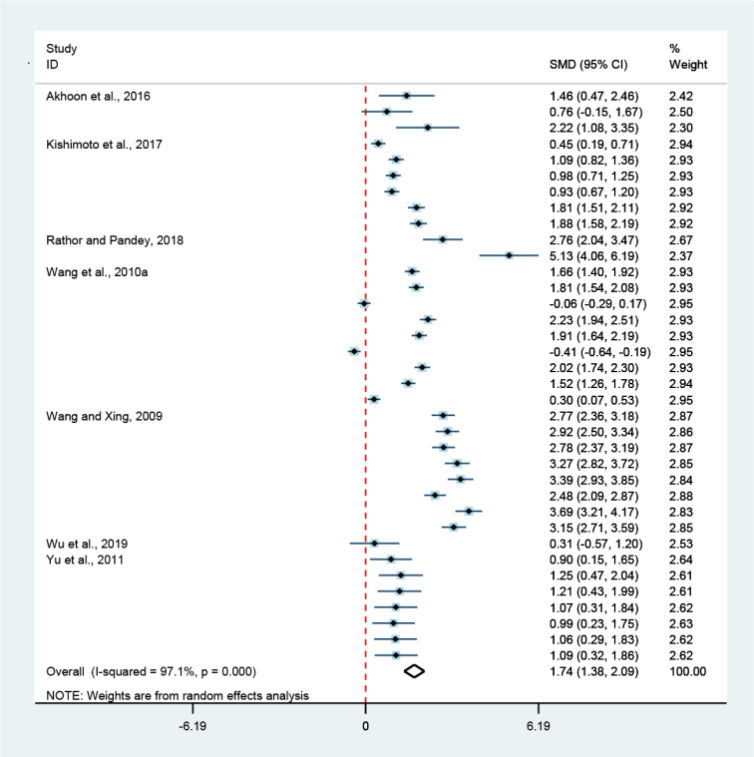
**Association of hormesis and pumping rate.** The diamond was the pooled effect size of pumping rate. The diamond did not intersect with the red-dotted-line, suggesting that hormesis could obviously enhance (on the right) or reduce (on the left) the pumping rate of *C. elegans* (*p* < 0.05).

**Figure 5:**
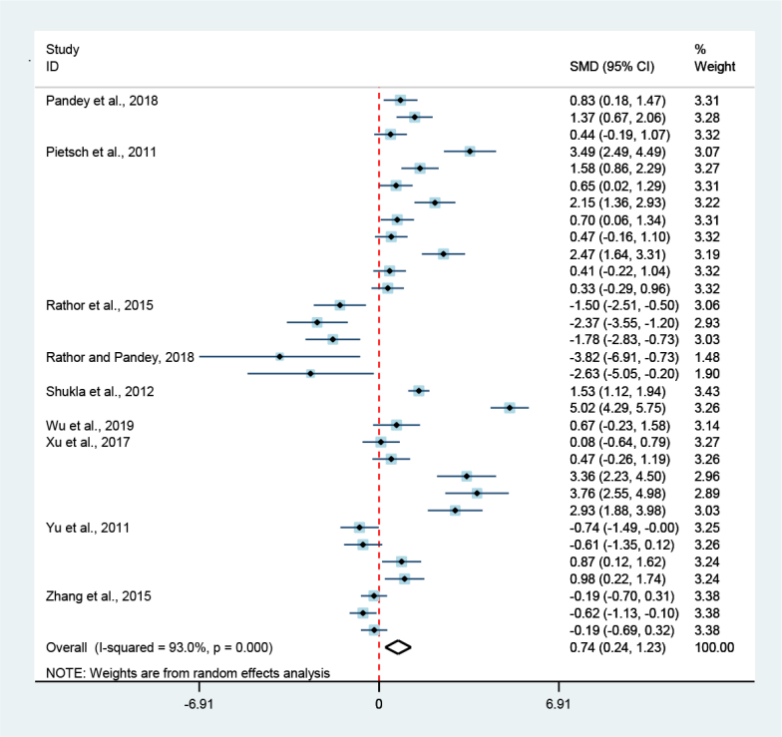
**Association of hormesis and lipofuscin level.** The combined effect size of lipofuscin level was shown by the diamond. The diamond did not intersect with red-dotted-line, indicating that hormesis could significantly increase (on the right) or decrease (on the left) the lipofuscin level of *C. elegans* (*p* < 0.05).

**Figure 6:**
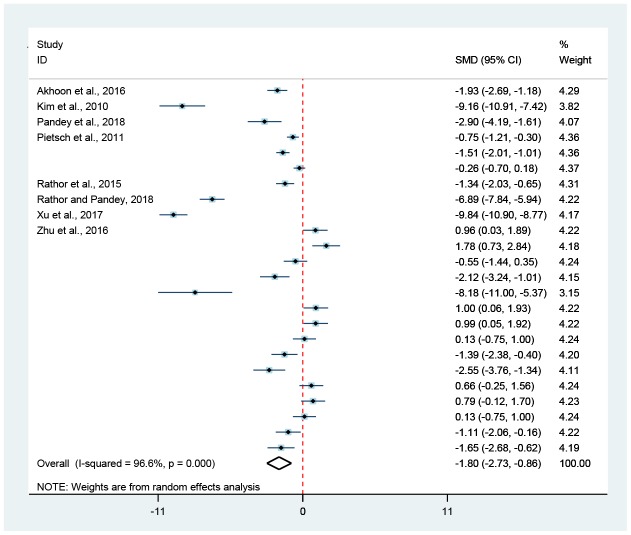
**Association of hormesis and stress resistance.** The diamond showed the pooled effect size of stress resistance, which did not intersect with the red-dotted-line, indicating hormesis could obviously increase (on the right) or decrease (on the left) the stress resistance of *C. elegans* (*p* < 0.05).

**Figure 7:**
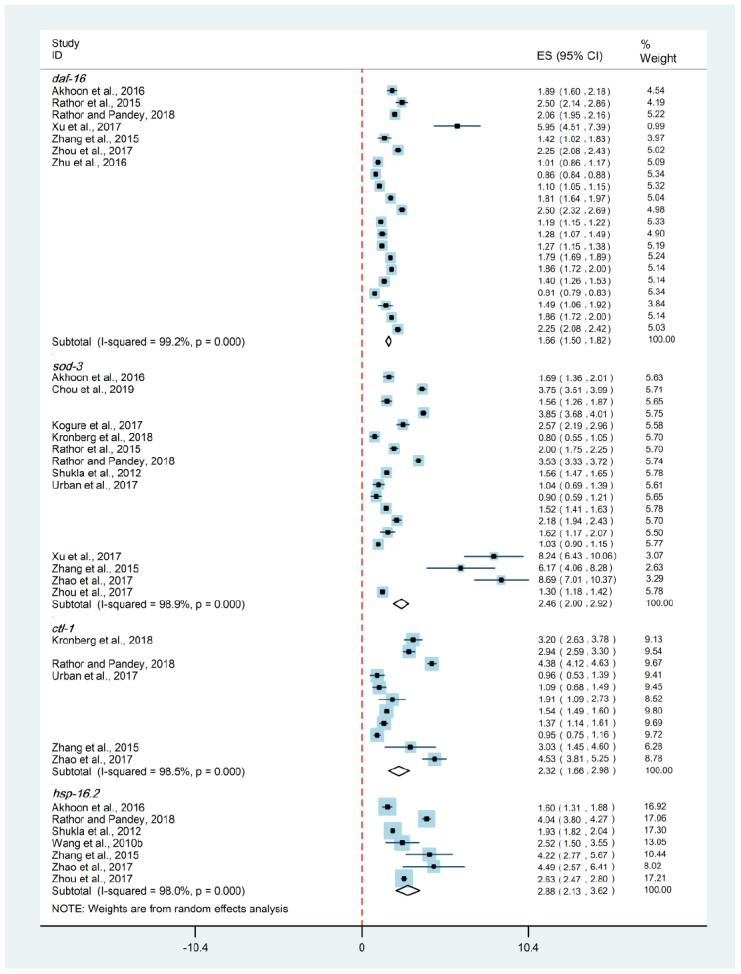
**Relative expression levels of Dauer formation protein-16-related genes.** The diamond indicated the combined effect size of DAF-16-related genes, which did not intersect with the red-dotted-line, indicating hormesis could obviously increase (on the right) or decrease (on the left) the expression of these genes (*p* < 0.05). Abbreviation: *daf-16*, *dauer formation protein-16*; *sod-3*, *superoxide dismutase-3*; *ctl-1*, *catalase-1*; *hsp-16.2*, *small heat shock protein-16.2*.

The combinations of relevant effect size estimates were summarized in Figure 1. Substantial heterogeneities were observed among included studies (*p* < 0.01), due to the exploratory nature of animal studies (Figures 2–7). Further sensitivity analysis showed that the meta-analytic results were stable and unchanged (Supplementary Figure 1). Moreover, Egger’s test indicated that there was no obvious publication bias except the indicator of bend frequency (Supplementary Figure 2).

### Hormesis and mean lifespan

This meta-analysis indicated that hormetic effect could significantly extend the mean lifespan of wild-type worms, with an increase of 16.7% (Random-effect model; SMD = 1.24, 95% CIs = 0.77, 1.70; *p* < 0.05) (Figures 1A and 2) under normal culture condition and 25.1% (Random-effect model; SMD = 1.94, 95% CIs = 0.93, 2.96; *p* < 0.05) (Figures 1E and 6) under stress culture condition.

### Hormesis and healthspan

Compared to untreated controls, hormesis could not only remarkably improve the bend frequency by 28.9% (Random-effect model; SMD = 1.74, 95% CIs = 1.38, 2.09; *p* < 0.05) (Figures 1B and 3) and the pumping rate by 7.0% (Random-effect model; SMD = 0.74, 95% CIs = 0.24, 1.23; *p* < 0.05) (Figures 1C and 4), but also obviously bring down the lipofuscin level by 15.9% (Random-effect model; SMD = -1.80, 95% CIs = -2.73, -0.86; *p* < 0.05) (Figures 1D and 5).

### Hormesis and DAF-16-related genes

To further explore the underlying mechanisms of hormesis, the mRNA levels of *daf-16* and its targeted genes were evaluated including *superoxide dismutase-3* (*sod-3*), *catalase-1* (*ctl-1*) and *small heat shock protein-16.2* (*hsp-16.2*). Compared with control groups, the transcript levels of these genes were increased up to 1.66-, 2.46-, 2.32- and 2.88-folds in hormesis groups (Random-effect model; *p* < 0.05), respectively (Figure 7).

## DISCUSSION

Lifespan, as a precisely calculable and highly credible parameter, has proven to be an excellent proxy for measuring the rate of aging [23, 24]. In this study, the lifespan-prolonging effect of hormesis under normal culture condition directly reflected the anti-aging effect of hormesis at the individual level. Consistently, the lifespan-prolonging effect of hormesis was also identified in other model organisms. Yu et al [25] reported that hyperbaric normoxia-induced (2 atm absolute pressure with 10% O_2_) hormesis could extend the mean lifespan in *Drosophila melanogaster* by 12% to 14%. Musa et al [26] denoted that hormetic effect triggered by heat shock (34 °C for 3 h) elongated the median replicative lifespan of budding yeast by 50%. More importantly, in mammalian rats, moderate exercise-induced hormesis prolonged the median lifespan by 9% to 19% [27]. However, it was worthy to note that the lifespan extension effect of hormesis was tightly associated with the genetic background in *D. melanogaster* [28]. In other words, the lifespan-prolonging in one line could be lifespan-limiting in other lines [28]. In the current study, only wild-type nematodes were included, thus effectively preventing the influence of genetic background on the meta-analytic results.

Healthspan describes the length of health life before the age-associated decline, which is species-specific and hardly defined [23, 29]. Based on the characteristics of *C. elegans*, three quantified indicators, including bend frequency, pumping rate and lipofuscin level, were selected as the representations to determine the effect of hormesis on healthspan [23]. Specifically, bend frequency was in line with neurotoxicity level [30], pumping rate suggested food intake ability [31], and lipofuscin level reflected intensity of lipid peroxidation [32]. In this study, sufficient evidence for the healthspan-enhancing effect of hormesis was observed, in which hormesis not only significantly improved the bend frequency and the pumping rate of *C. elegans*, but also obviously reduced the lipofuscin level. The positive role of hormesis in healthspan indicated the possibility of applying hormesis in improving healthy aging in *C. elegans*.

Stress resistance is an integral component of aging studies, and the ability of *C. elegans* to resist the external and internal perils is generally correlated with the length of lifespan [23]. In this study, the stress resistance is corresponding to the mean lifespan of *C. elegans* under stress (e.g. oxidation, heat, cold or UV-irradiation) culture condition. Some aging theories constructed a framework, proposing that the enhanced stress resistance was a direct cause of lifespan extension, like the free radical theory of aging [23, 33, 34]. The positive connection between stress resistance and lifespan was vividly manifested in this study. The significant prolongation of mean lifespan of *C. elegans* in hormesis group under stress culture condition suggested the resistance-increasing effect of hormesis. Consistently, the improvement in resilience of hormesis has also been identified in some clinical trials. For example, the low-dose radiation was effective for the treatment of inflammations [35], and repeated moderate exercise strengthened anti-oxidative defense [36]. As commented by Gems et al [37], hormesis does not kill us, just makes us stronger.

In *C. elegans*, DAF-16 is the major downstream target of the insulin/IGF-1 signaling pathway (IIS) that acts as the forkhead Box O (FoxO) transcription factor regulating lifespan and resistance [38, 39]. In this study, the mRNA levels of *daf-16* and its targeted genes were evaluated including *sod-3*, *ctl-1* that both conduced to the oxidative stress response, and *hsp-16.2* that contributed to heat shock response [38, 40–42]. The obviously increased expressions of these genes were highly corresponding to longer lifespan and higher resistance among hormetic nematodes, suggesting that the lifespan-prolonging and resistance-increasing effects of hormesis were mediated through *daf-16* activation. Moreover, Blagosklonny [18] proposed that aging was driven by over-activated signal-transduction pathways (e.g. Target of Rapamycin (TOR)) instead of the accumulation of molecular damage. If so, the anti-aging effect of hormesis could be perfectly explained.

Overall, in *C. elegans*, strong evidence for the anti-aging effect of hormesis was identified. The lifespan-prolonging, healthspan-enhancing and resistance-increasing effects of hormesis revealed the anti-aging role of hormesis at individual level, while the activated expression of DAF-16-related genes reflected the anti-aging role of hormesis at molecular level. Apparently, all of these involved indicators supported the application of hormesis in aging intervention in *C. elegans*. It is still unclear to what extent the applicability can be transferred from model organisms to humans [4, 11]. However, given that *daf-16* was highly conservative [43, 44], hormesis provided the theoretical possibility of delaying intrinsic aging through exogenous intervention among humans.

## CONCLUSIONS

The lifespan-prolonging, healthspan-enhancing and resistance-increasing effects of hormesis were identified in *C. elegans*. The consistently positive findings across different aging indicators demonstrated that hormesis was a promising modulator of aging. The significant activation of *daf-16* and its target genes, including *sod-3*, *ctl-1* and *hsp-16.2*, was one of the potential molecular mechanisms driving these beneficial effects of hormesis

## MATERIALS AND METHODS

### Proposal registration and searching strategy

The review protocol was registered in the International Prospective Register of Systematic Reviews (PROSPERO) with the reference number of CRD42019117838.

Three electronic databases were searched, including *Medline*, *Embase* and *the Cochrane Library*, using the combination keywords: (“hormesis” OR “hormetic” OR “biphasic” OR “preconditioning” OR “conditioning” OR “adaptive response” OR “acclimation response” OR “stress response” OR “dose response” OR “dose-response”) AND (“anti-ageing” OR “antiageing” OR “anti-aging” OR “antiaging” OR “ageing” OR “aging” OR “life” OR “lifespan” OR “extend” OR “extending” OR “extension” OR “prolong” OR “prolonging” OR “prolongation” OR “longevity” OR “survival” OR “resistance” OR “healthspan” OR “health span” OR “bend” OR “bending” OR “pump” OR “pumping” OR “lipofuscin” OR “pigment”) AND (“Caenorhabditis elegans” OR “C. elegans” OR “worm” OR “nematode”), publishing in English or Chinese. Additionally, gray literatures such as conference papers and references listed were also searched using the above keywords via *Index to Scientific and Technical Proceedings*, *Web of Science* and *Baidu Scholar*.

### Inclusion criteria

The titles, abstracts and keywords of all retrieved papers were preliminarily separately screened by two researchers (T. Sun and J. Zhan). Then, the full texts of potentially qualified papers were downloaded and independently reviewed for inclusion according to the following criteria: (i) original research paper; (ii) designed at least one control group and one hormesis group; (iii) the experimental animals were healthy *C. elegans*; (iv) reported at least one outcome induced by hormesis about aging/anti-aging in wild-type worms; (v) the sample size of each study and the mean value with standard deviation (*SD*)/standard error (*SE*) of each outcome were available. If a paper involving missing data, an inquiry email was sent to the correspondence author for the raw data. However, if the data were not available within one month, the paper would be excluded. Then cross-checking was conducted, with disagreements settled by discussion, or consultation with the third researchers. After the final selection, 198 effect size estimates (including 33 and 15 for mean lifespan under normal condition and stress condition respectively, 36 for bend frequency, 32 for pumping rate, 24 for lipofuscin level, and 58 for mRNA expression level of *dauer formation protein-16* (*daf-16*) and its target genes) involving 26 papers met the inclusion criteria, and were retained in this meta-analysis (see Supplementary Table 1).

### Data analysis

This meta-analysis was performed using the STATA v12.0 software (Stata Corporation, College Station, TX, USA). Chi-squared-based I^2^ test was conducted to evaluate the heterogeneity among studies, with the values I^2^ of 25%, 50% and 75% representing low, moderate and high degrees, respectively [45]. If the value of I^2^ was greater than 50%, the random-effect model based on the method proposed by DerSimonian and Laird would be adopted to account for the high heterogeneity among studies [46], followed by “leave-one-out” sensitivity analysis to test the robustness of meta-analytic results, or else, the fixed-effect model based on inverse variance method would be used [47]. Egger’s linear regression test would be used for quantitatively estimating the risk of publication bias [45]. The unbiased estimate of effect size was in accordance to Cohen’s standardized mean difference (SMD) with 95% confidence intervals (CIs) [45]. The significance level was set at *p* < 0.05.

## Supplementary Material

Supplementary Figures

Supplementary Table 1

## References

[1] Calabrese EJ, Baldwin LA. Defining hormesis. Hum Exp Toxicol. 2002; 21:91–97. 10.1191/0960327102ht217oa 12102503

[2] Calabrese EJ. The emergence of the dose-response concept in biology and medicine. Int J Mol Sci. 2016; 17:2034. 10.3390/ijms17122034 27929392 PMC5187834

[3] Calabrese EJ, Iavicoli I, Calabrese V. Hormesis: why it is important to biogerontologists. Biogerontology. 2012; 13:215–35. 10.1007/s10522-012-9374-7 22270337

[4] Rattan SI. Hormesis for healthy aging. In: Rattan SI, Kyriazis M, editors. The science of hormesis in health and longevity. UK: Academic Press; 2019 pp. 201–12. 10.1016/B978-0-12-814253-0.00018-8

[5] Calabrese EJ, Mattson MP. How does hormesis impact biology, toxicology, and medicine? NPJ Aging Mech Dis. 2017; 3:13. 10.1038/s41514-017-0013-z 28944077 PMC5601424

[6] Rattan SI. Hormesis in aging. Ageing Res Rev. 2008; 7:63–78. 10.1016/j.arr.2007.03.002 17964227

[7] Blagosklonny MV. Aging: ROS or TOR. Cell Cycle. 2008; 7:3344–54. 10.4161/cc.7.21.6965 18971624

[8] Russell EG, Cotter TG. New insight into the role of reactive oxygen species (ROS) in cellular signal-transduction processes. Int Rev Cell Mol Biol. 2015; 319:221–54. 10.1016/bs.ircmb.2015.07.004 26404470

[9] McClure CD, Zhong W, Hunt VL, Chapman FM, Hill FV, Priest NK. Hormesis results in trade-offs with immunity. Evolution. 2014; 68:2225–33. 10.1111/evo.12453 24862588 PMC4282086

[10] Le Bourg É, Rattan SI. Hormesis and trade-offs: a comment. Dose Response. 2014; 12:522–24. 10.2203/dose-response.14-054.LeBourg 25552954 PMC4267446

[11] Le Bourg É. Mild stress-induced hormesis. In: Rattan SI, Kyriazis M, editors. The science of hormesis in health and longevity. UK: Academic Press; 2019 pp. 25–33. 10.1016/B978-0-12-814253-0.00002-4

[12] Calabrese EJ, Dhawan G, Kapoor R, Iavicoli I, Calabrese V. Hormesis: a fundamental concept with widespread biological and biomedical applications. Gerontology. 2016; 62:530–35. 10.1159/000441520 26535577

[13] Rattan SI, Kryzch V, Schnebert S, Perrier E, Nizard C. Hormesis-based anti-aging products: a case study of a novel cosmetic. Dose Response. 2013; 11:99–108. 10.2203/dose-response.11-054.Rattan 23548988 PMC3578457

[14] Cui J, Yang G, Pan Z, Zhao Y, Liang X, Li W, Cai L. Hormetic response to low-dose radiation: focus on the immune system and its clinical implications. Int J Mol Sci. 2017; 18:E280. 10.3390/ijms18020280 28134809 PMC5343816

[15] Mägdefrau AS, Ludwig K, Weigel C, Köse N, Guerra GM, Dakhovnik A, Kosan C. DNA-damage-induced hormetic responses. In: Rattan SI, Kyriazis M, editors. The science of hormesis in health and longevity. UK: Academic Press; 2019 pp. 149–59. 10.1016/B978-0-12-814253-0.00013-9

[16] Wang L, Zou W, Zhong Y, An J, Zhang X, Wu M, Yu Z. The hormesis effect of BDE-47 in HepG2 cells and the potential molecular mechanism. Toxicol Lett. 2012; 209:193–201. 10.1016/j.toxlet.2011.12.014 22233939

[17] López-Otín C, Blasco MA, Partridge L, Serrano M, Kroemer G. The hallmarks of aging. Cell. 2013; 153:1194–217. 10.1016/j.cell.2013.05.039 23746838 PMC3836174

[18] Blagosklonny MV. Hormesis does not make sense except in the light of TOR-driven aging. Aging (Albany NY). 2011; 3:1051–62. 10.18632/aging.100411 22166724 PMC3249451

[19] Gurevitch J, Koricheva J, Nakagawa S, Stewart G. Meta-analysis and the science of research synthesis. Nature. 2018; 555:175–82. 10.1038/nature25753 29517004

[20] Liu H, Bravata DM, Olkin I, Nayak S, Roberts B, Garber AM, Hoffman AR. Systematic review: the safety and efficacy of growth hormone in the healthy elderly. Ann Intern Med. 2007; 146:104–15. 10.7326/0003-4819-146-2-200701160-00005 17227934

[21] Peterson MD, Rhea MR, Sen A, Gordon PM. Resistance exercise for muscular strength in older adults: a meta-analysis. Ageing Res Rev. 2010; 9:226–37. 10.1016/j.arr.2010.03.004 20385254 PMC2892859

[22] Johnson TE. Advantages and disadvantages of *Caenorhabditis elegans* for aging research. Exp Gerontol. 2003; 38:1329–32. 10.1016/j.exger.2003.10.020 14698813

[23] Keith SA, Amrit FR, Ratnappan R, Ghazi A. The *C. elegans* healthspan and stress-resistance assay toolkit. Methods. 2014; 68:476–86. 10.1016/j.ymeth.2014.04.003 24727065

[24] Kenyon CJ. The genetics of ageing. Nature. 2010; 464:504–12. 10.1038/nature08980 20336132

[25] Yu S, Lee E, Tsogbadrakh B, Son GI, Kim M. Prenatal hyperbaric normoxia treatment improves healthspan and regulates chitin metabolic genes in *Drosophila melanogaster.* Aging (Albany NY). 2016; 8:2538–50. 10.18632/aging.101084 27777382 PMC5115905

[26] Musa M, Perić M, Bou Dib P, Sobočanec S, Šarić A, Lovrić A, Rudan M, Nikolić A, Milosević I, Vlahoviček K, Raimundo N, Kriško A. Heat-induced longevity in budding yeast requires respiratory metabolism and glutathione recycling. Aging (Albany NY). 2018; 10:2407–27. 10.18632/aging.101560 30227387 PMC6188503

[27] Navarro A, Gomez C, López-Cepero JM, Boveris A. Beneficial effects of moderate exercise on mice aging: survival, behavior, oxidative stress, and mitochondrial electron transfer. Am J Physiol Regul Integr Comp Physiol. 2004; 286:R505–11. 10.1152/ajpregu.00208.2003 14615275

[28] Sarup P, Loeschcke V. Life extension and the position of the hormetic zone depends on sex and genetic background in *Drosophila melanogaster.* Biogerontology. 2011; 12:109–17. 10.1007/s10522-010-9298-z 20711813

[29] Tissenbaum HA. Genetics, life span, health span, and the aging process in *Caenorhabditis elegans.* J Gerontol A Biol Sci Med Sci. 2012; 67:503–10. 10.1093/gerona/gls088 22499764 PMC3410663

[30] Wang D, Xing X. Assessment of locomotion behavioral defects induced by acute toxicity from heavy metal exposure in nematode *Caenorhabditis elegans.* J Environ Sci (China). 2008; 20:1132–37. 10.1016/S1001-0742(08)62160-9 19143322

[31] Pandey S, Tiwari S, Kumar A, Niranjan A, Chand J, Lehri A, Chauhan PS. Antioxidant and anti-aging potential of Juniper berry (*Juniperus communis* L.) essential oil in *Caenorhabditis elegans* model system. Ind Crops Prod. 2018; 120:113–22. 10.1016/j.indcrop.2018.04.066

[32] Leomanni A, Schettino T, Calisi A, Gorbi S, Mezzelani M, Regoli F, Lionetto MG. Antioxidant and oxidative stress related responses in the Mediterranean land snail Cantareus apertus exposed to the carbamate pesticide Carbaryl. Comp Biochem Physiol C Toxicol Pharmacol. 2015; 168:20–27. 10.1016/j.cbpc.2014.11.003 25451076

[33] Harman D. Aging: a theory based on free radical and radiation chemistry. J Gerontol. 1956; 11:298–300. 10.1093/geronj/11.3.298 13332224

[34] Johnson TE, Lithgow GJ, Murakami S. Hypothesis: interventions that increase the response to stress offer the potential for effective life prolongation and increased health. J Gerontol A Biol Sci Med Sci. 1996; 51:B392–95. 10.1093/gerona/51A.6.B392 8914487

[35] Kojima S, Tsukimoto M, Shimura N, Koga H, Murata A, Takara T. Treatment of cancer and inflammation with low-dose ionizing radiation: three case reports. Dose Response. 2017; 15:1559325817697531. 10.1177/1559325817697531 28539853 PMC5433552

[36] Radak Z, Chung HY, Koltai E, Taylor AW, Goto S. Exercise, oxidative stress and hormesis. Ageing Res Rev. 2008; 7:34–42. 10.1016/j.arr.2007.04.004 17869589

[37] Gems D, Partridge L. Stress-response hormesis and aging: “that which does not kill us makes us stronger”. Cell Metab. 2008; 7:200–03. 10.1016/j.cmet.2008.01.001 18316025

[38] Zhang J, Lu L, Zhou L. Oleanolic acid activates daf-16 to increase lifespan in *Caenorhabditis elegans.* Biochem Biophys Res Commun. 2015; 468:843–49. 10.1016/j.bbrc.2015.11.042 26592451

[39] Jensen VL, Gallo M, Riddle DL. Targets of DAF-16 involved in *Caenorhabditis elegans* adult longevity and dauer formation. Exp Gerontol. 2006; 41:922–27. 10.1016/j.exger.2006.06.058 17055208

[40] Murphy CT, McCarroll SA, Bargmann CI, Fraser A, Kamath RS, Ahringer J, Li H, Kenyon C. Genes that act downstream of DAF-16 to influence the lifespan of Caenorhabditis elegans. Nature. 2003; 424:277–83. 10.1038/nature01789 12845331

[41] Hartwig K, Heidler T, Moch J, Daniel H, Wenzel U. Feeding a ROS-generator to *Caenorhabditis elegans* leads to increased expression of small heat shock protein HSP-16.2 and hormesis. Genes Nutr. 2009; 4:59–67. 10.1007/s12263-009-0113-x 19252938 PMC2654055

[42] Kronberg MF, Clavijo A, Moya A, Rossen A, Calvo D, Pagano E, Munarriz E. Glyphosate-based herbicides modulate oxidative stress response in the nematode *Caenorhabditis elegans.* Comp Biochem Physiol C Toxicol Pharmacol. 2018; 214:1–8. 10.1016/j.cbpc.2018.08.002 30142450

[43] van der Horst A, Burgering BM. Stressing the role of FoxO proteins in lifespan and disease. Nat Rev Mol Cell Biol. 2007; 8:440–50. 10.1038/nrm2190 17522590

[44] Wang X, Zhang J, Lu L, Zhou L. The longevity effect of echinacoside in *Caenorhabditis elegans* mediated through daf-16. Biosci Biotechnol Biochem. 2015; 79:1676–83. 10.1080/09168451.2015.1046364 26027643

[45] Higgins JP, Green S. Cochrane handbook for systematic reviews of interventions. 2011 http://handbook.cochrane.org

[46] DerSimonian R, Laird N. Meta-analysis in clinical trials. Control Clin Trials. 1986; 7:177–88. 10.1016/0197-2456(86)90046-2 3802833

[47] Deeks JJ, Altman D, Bradburn MJ. Statistical methods for examining heterogeneity and combining results from several studies in meta-analysis. In: Egger M, Smith GD, Altman DG, editors. Systematic reviews in health care: meta-analysis in context. UK: BMJ Publishing Group; 2001 pp. 285–312. 10.1002/9780470693926.ch15

