# Supplementary material for: Meta-analytic evidence for the anti-aging effect of hormesis on Caenorhabditis elegans
Source: Aging (Albany NY). 2020 Feb 7;12(3):2723–46. doi: 10.18632/aging.102773 (PMC7041774; doi:10.18632/aging.102773)
Supplement: Supplementary Figures [file aging-12-102773-s002..pdf]

SUPPLEMENTARY FIGURES WITH REFERENCES

A

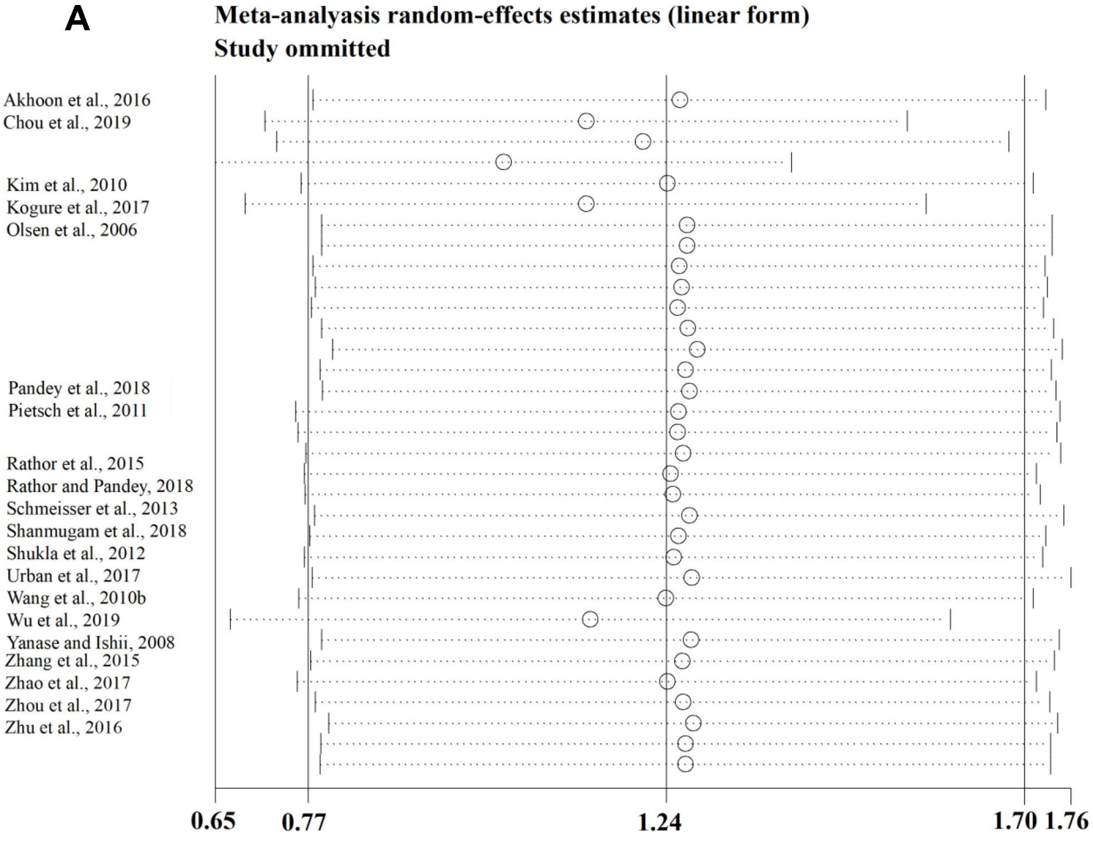

B

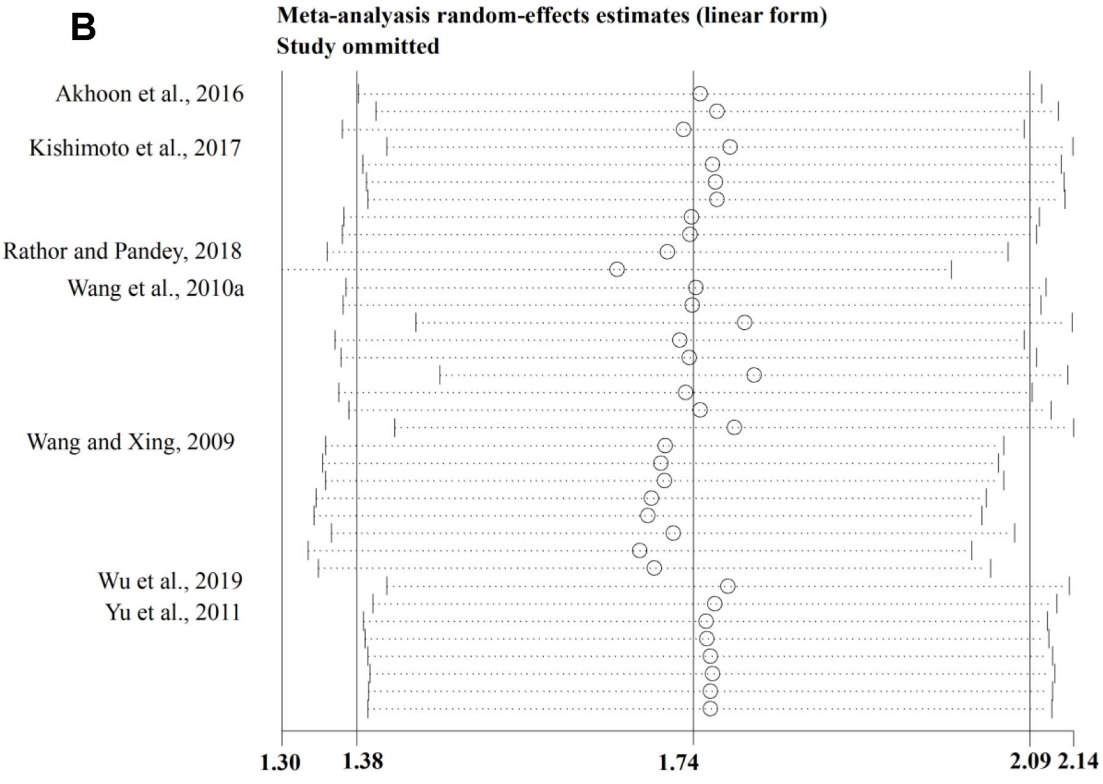

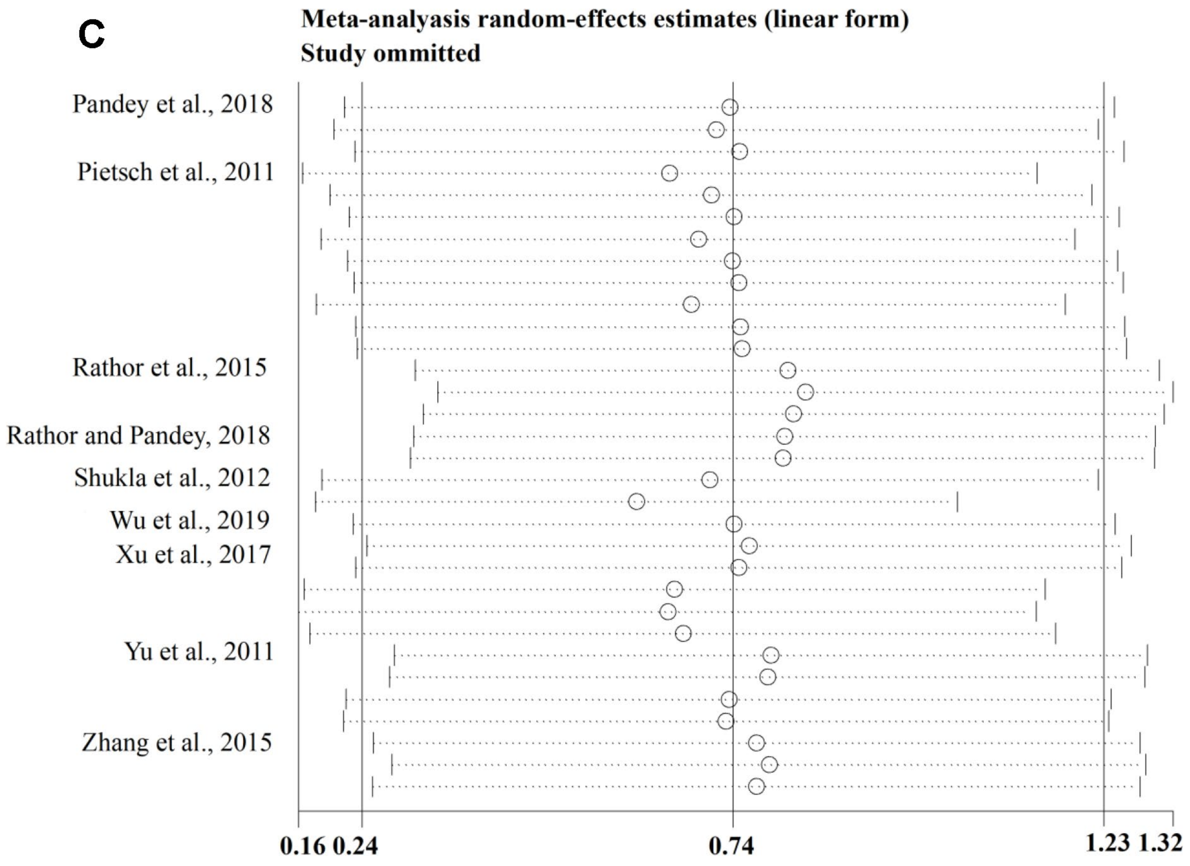
C
Meta-analaysis random-effects estimates (linear form)
Study ommitted
Pandey et al., 2018
Pietsch et al., 2011
Rathor et al., 2015
Rathor and Pandey, 2018
Shukla et al., 2012
Wu et al., 2019
Xu et al., 2017
Yu et al., 2011
Zhang et al., 2015
0.16 0.24
0.74
1.23 1.32

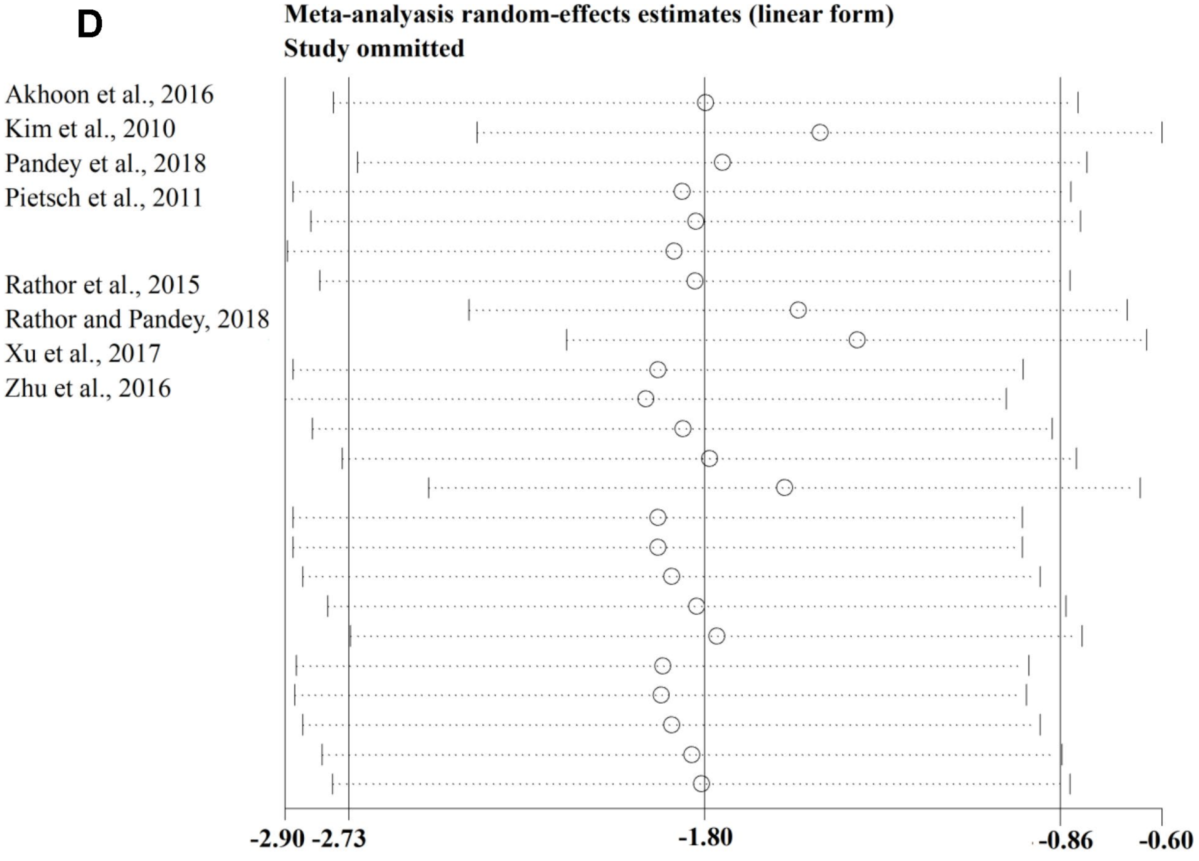
D
Meta-analaysis random-effects estimates (linear form)
Study ommitted
Akhoon et al., 2016
Kim et al., 2010
Pandey et al., 2018
Pietsch et al., 2011
Rathor et al., 2015
Rathor and Pandey, 2018
Xu et al., 2017
Zhu et al., 2016
-2.90 -2.73
-1.80
-0.86
-0.60

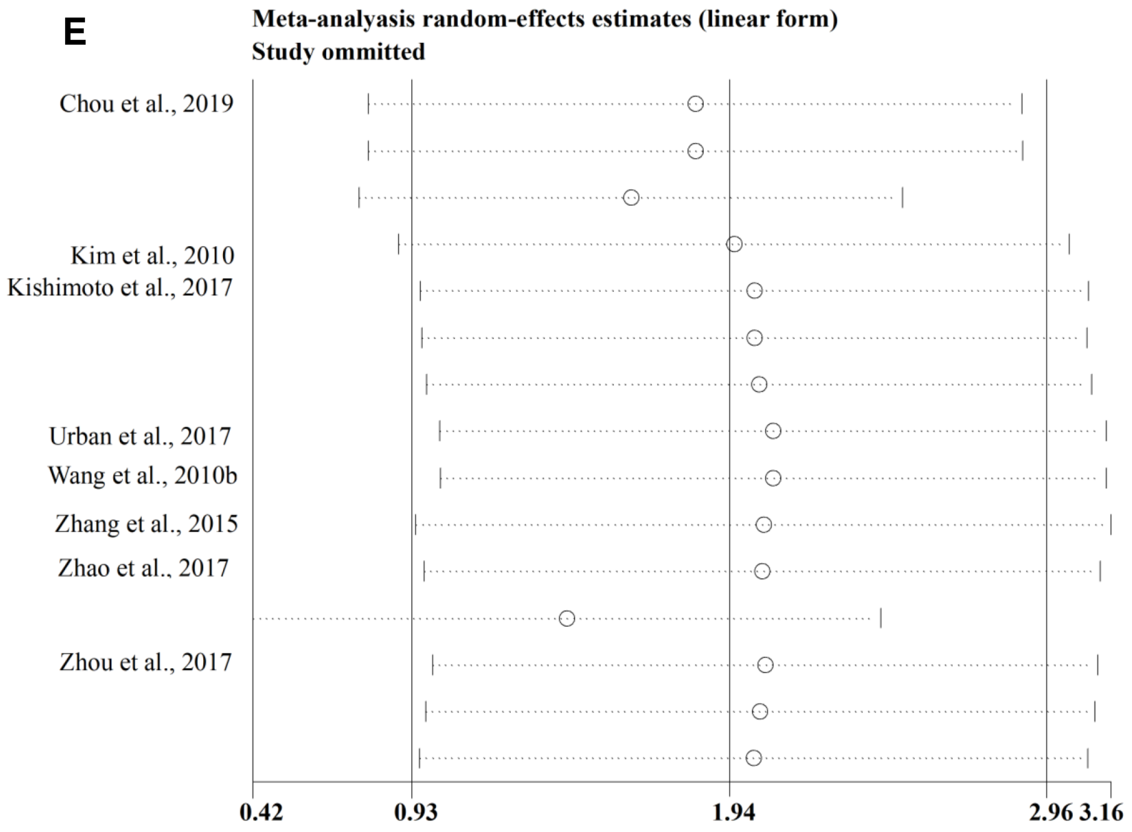

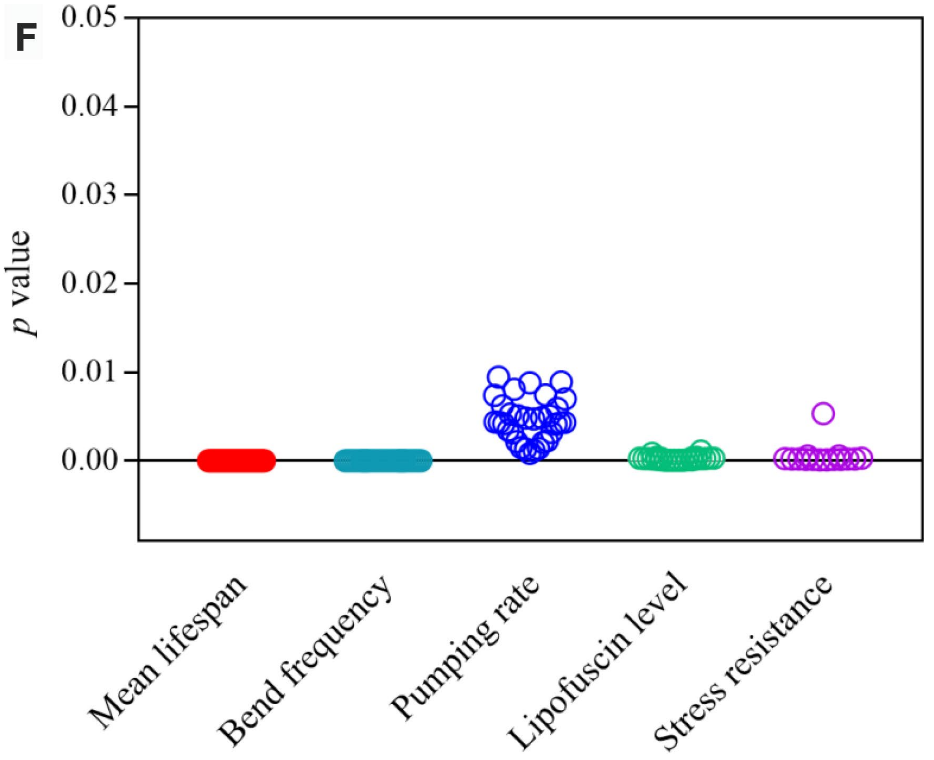

Supplementary Figure 1. "Leave-one-out" sensitivity analyses. (**A**–**E**) denoted the "leave-one-out" sensitivity analyses of mean lifespan, bend frequency, pumping rate, lipofuscin level and stress resistance, respectively. (**F**) was the summary of the significant re-test after omitting one study. As shown in (**F**), the meta-analytic results of all indicators were stable.

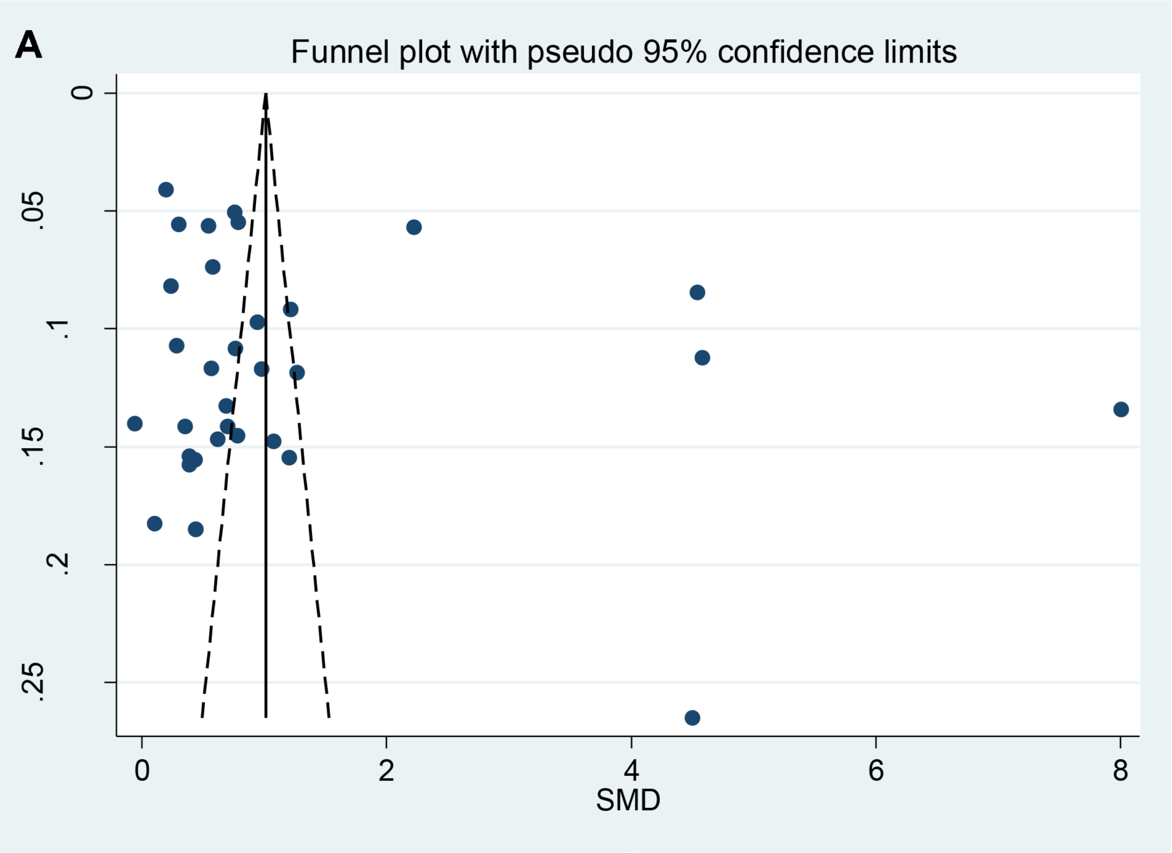

A
Funnel plot with pseudo 95% confidence limits
0
.05
.1
.15
.2
.25
0
2
4
6
8
SMD

I

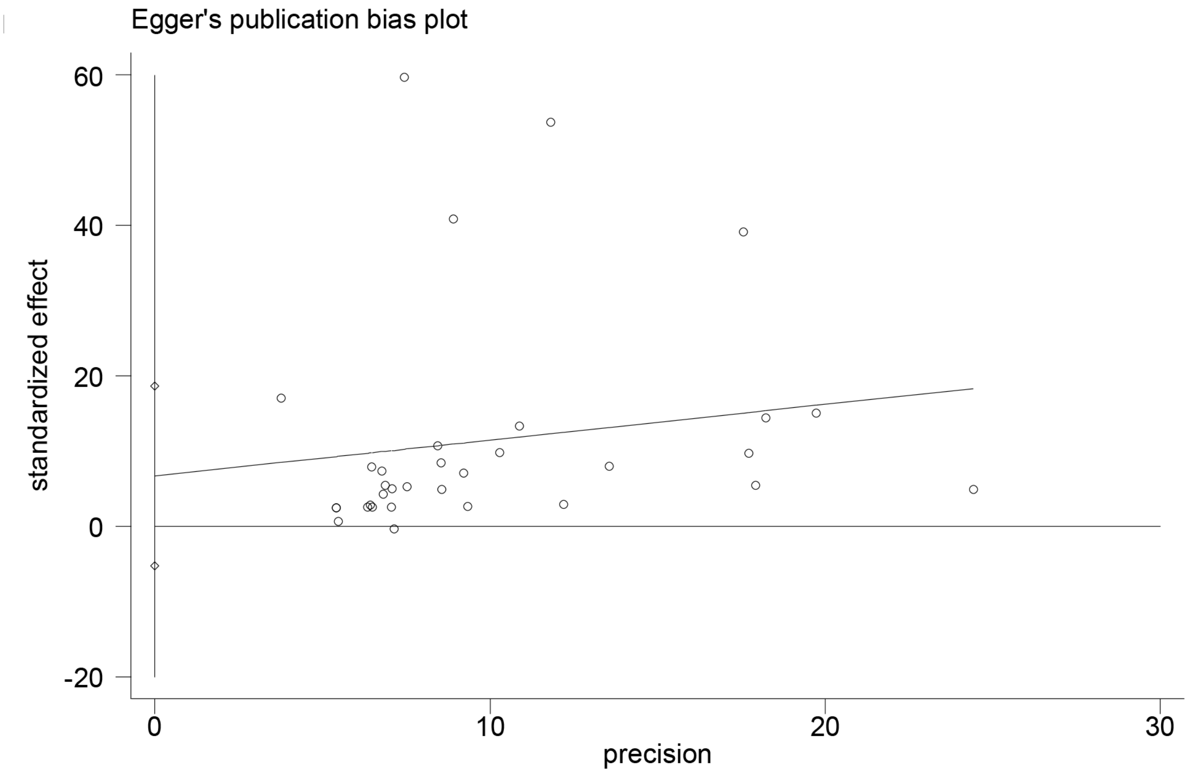

Egger's publication bias plot
60
40
20
0
-20
standardized effect
0
10
20
30
precision

II

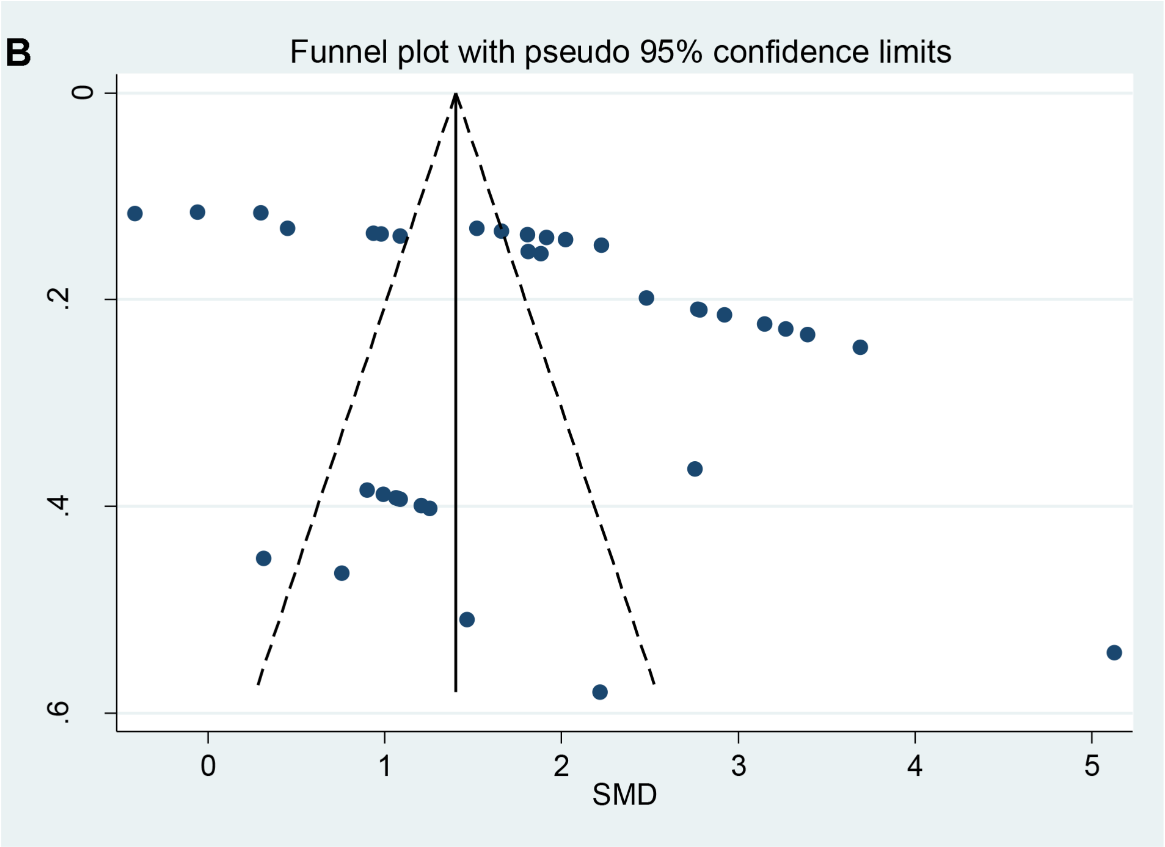
B
Funnel plot with pseudo 95% confidence limits
0
.2
.4
.6
0
1
2
3
4
5
SMD

I

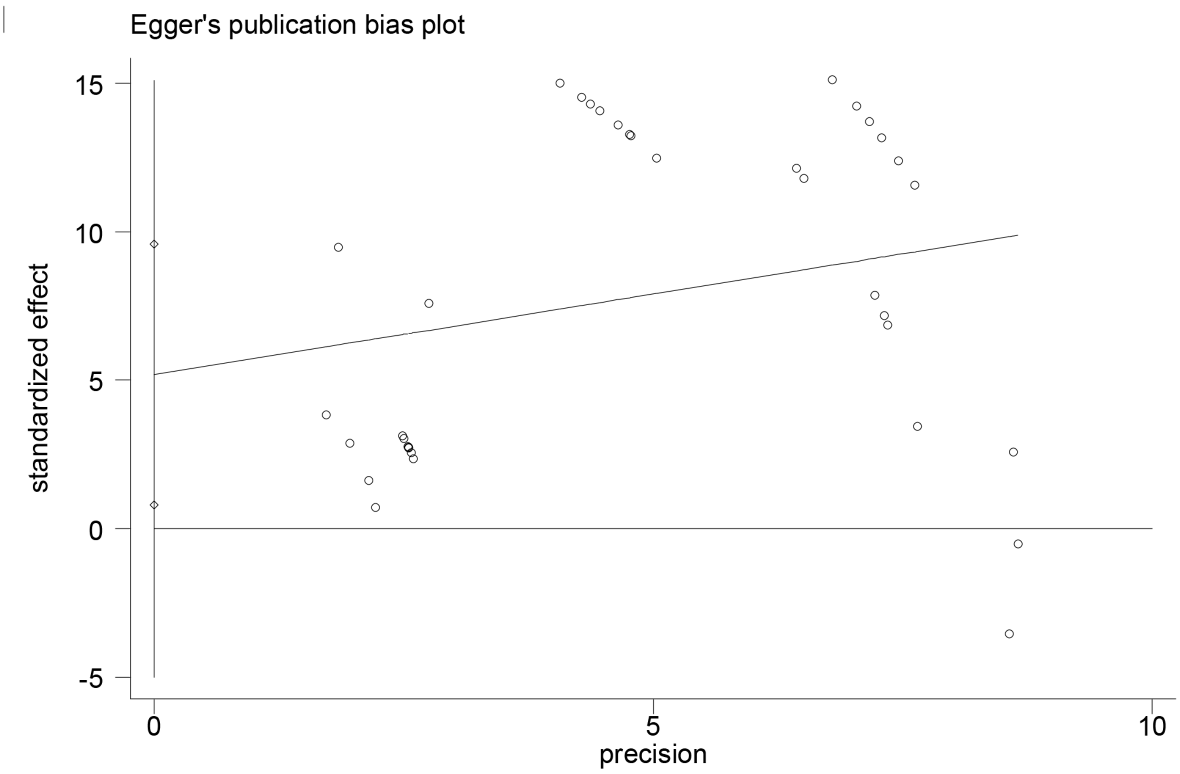
Egger's publication bias plot
15
10
5
0
-5
standardized effect
0
5
10
precision

II

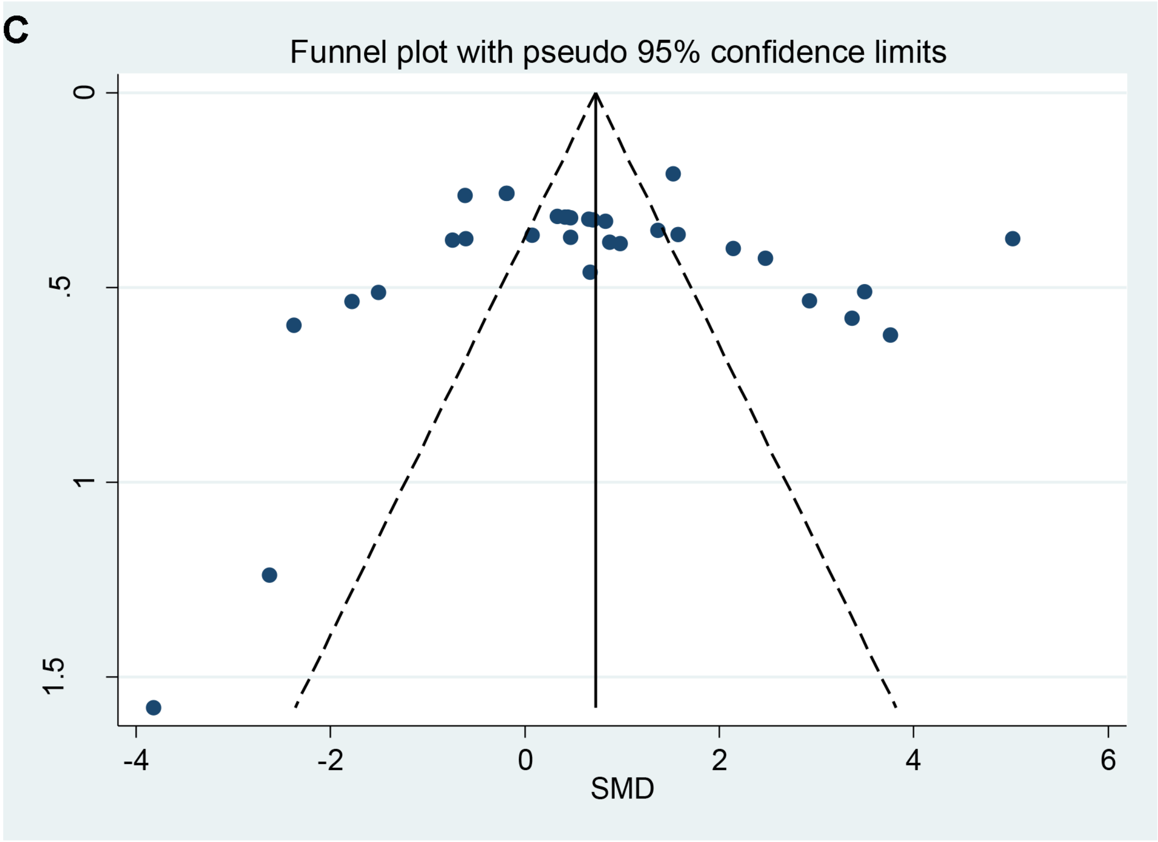
C
Funnel plot with pseudo 95% confidence limits
0
.5
1
1.5
-4
-2
0
2
4
6
SMD

I

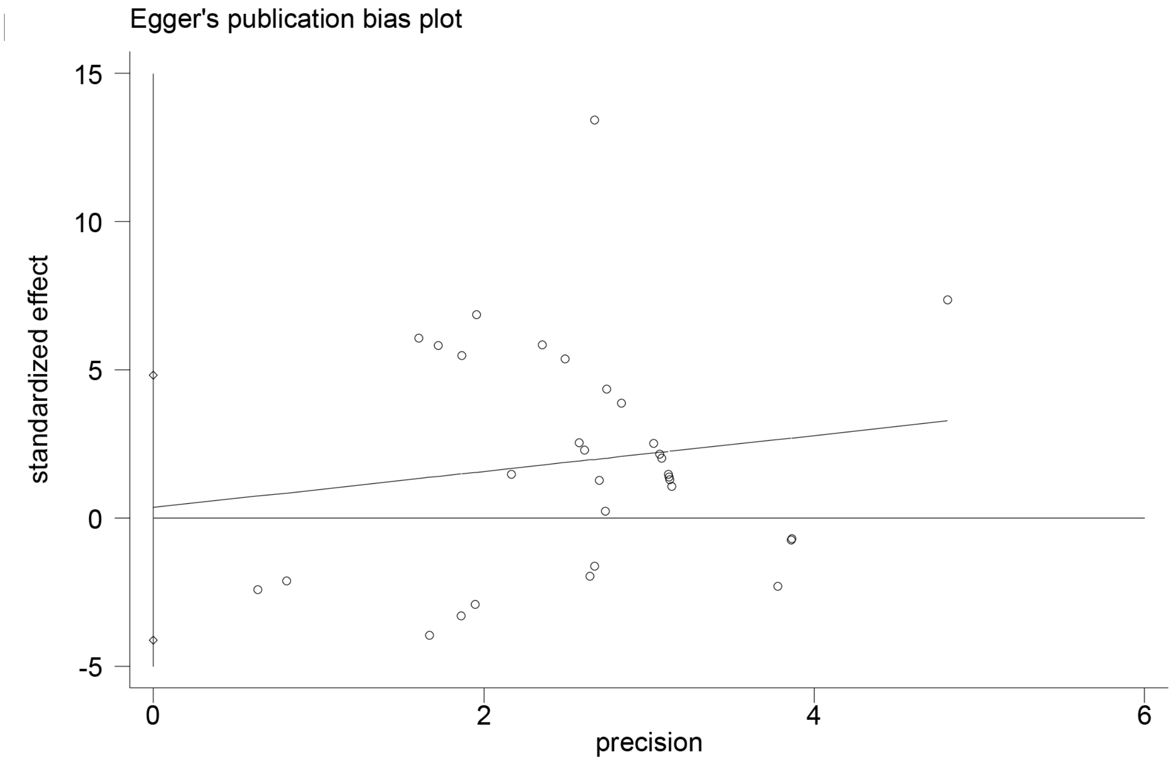
Egger's publication bias plot
standardized effect
15
10
5
0
-5
0
2
4
6
precision

II

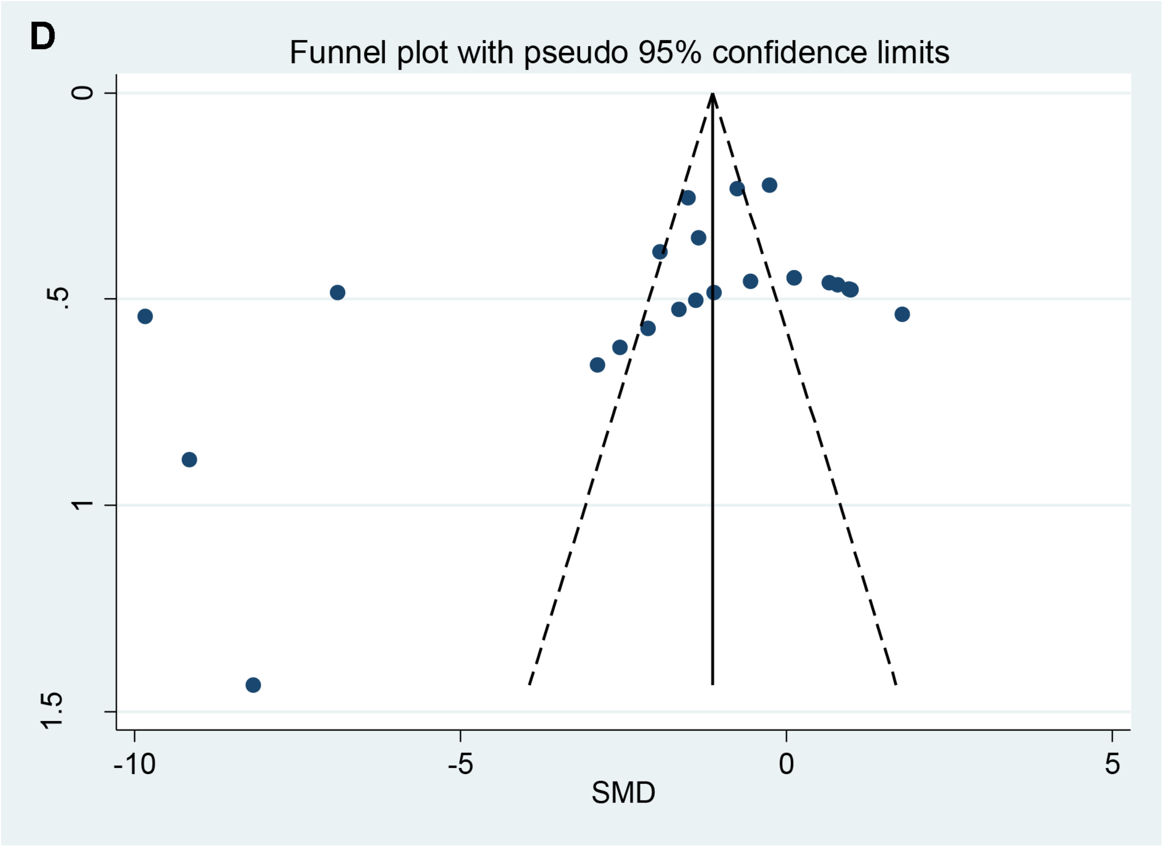
D
Funnel plot with pseudo 95% confidence limits
0
.5
1
1.5
-10
-5
0
5
SMD

I

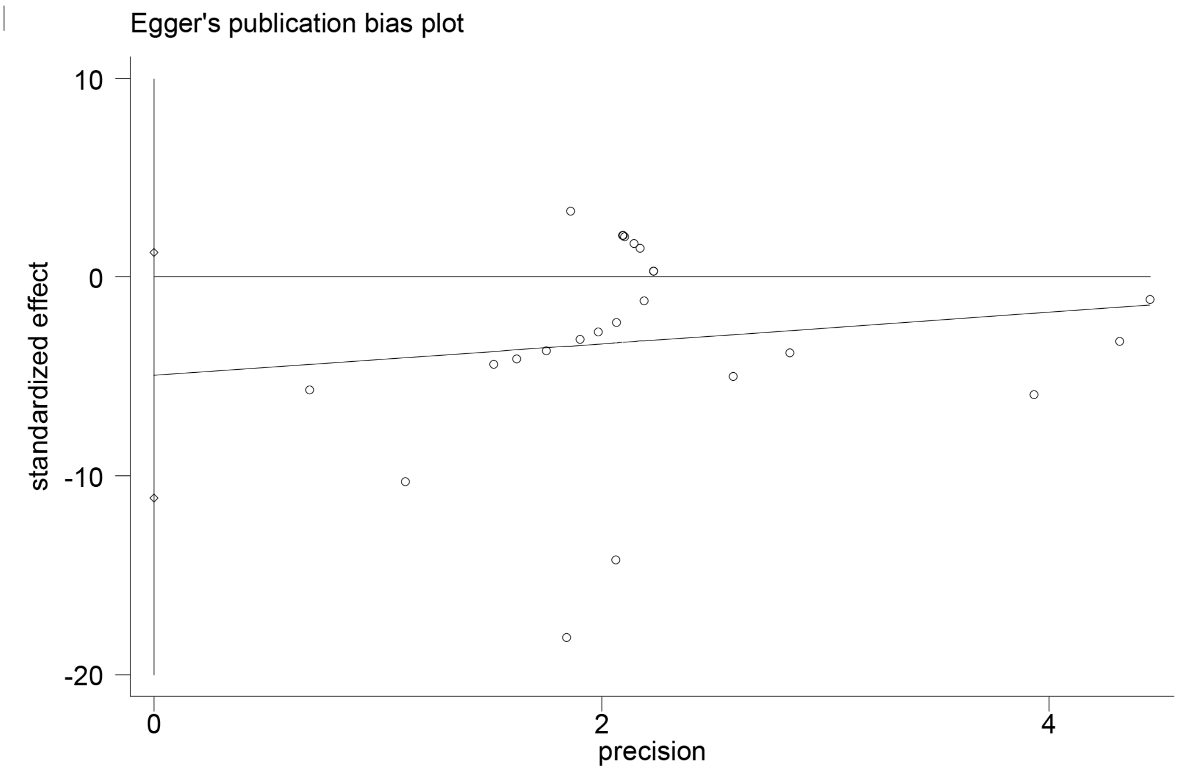
Egger's publication bias plot
10
0
-10
-20
standardized effect
0
2
4
precision

II

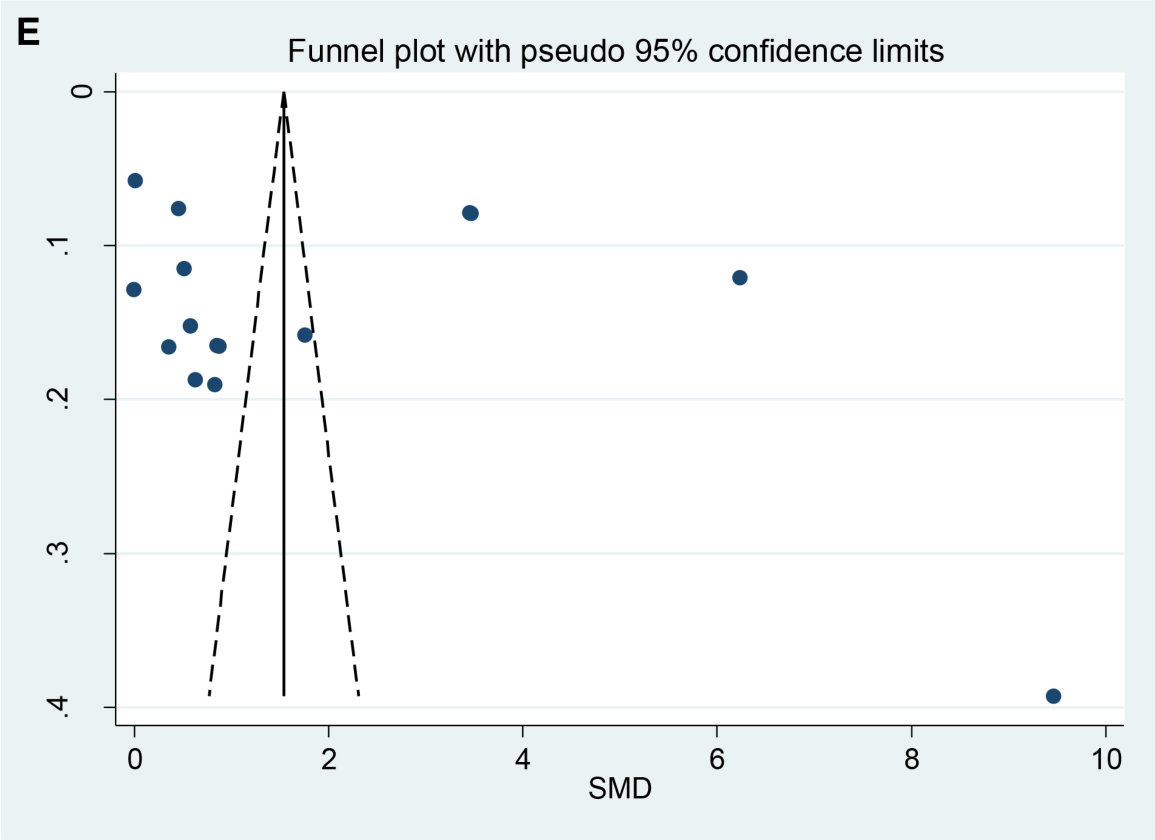
E
Funnel plot with pseudo 95% confidence limits
0
.1
.2
.3
.4
0
2
4
6
8
10
SMD

I

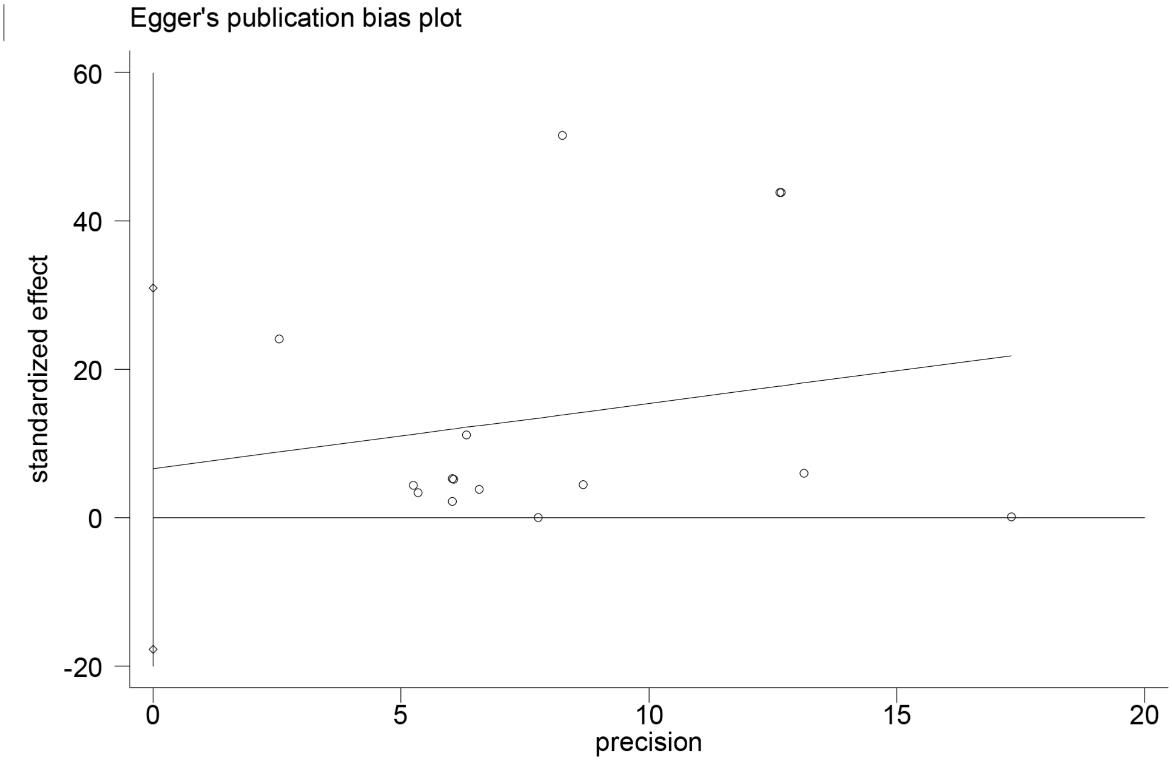
Egger's publication bias plot
60
40
20
0
-20
standardized effect
0
5
10
15
20
precision

II

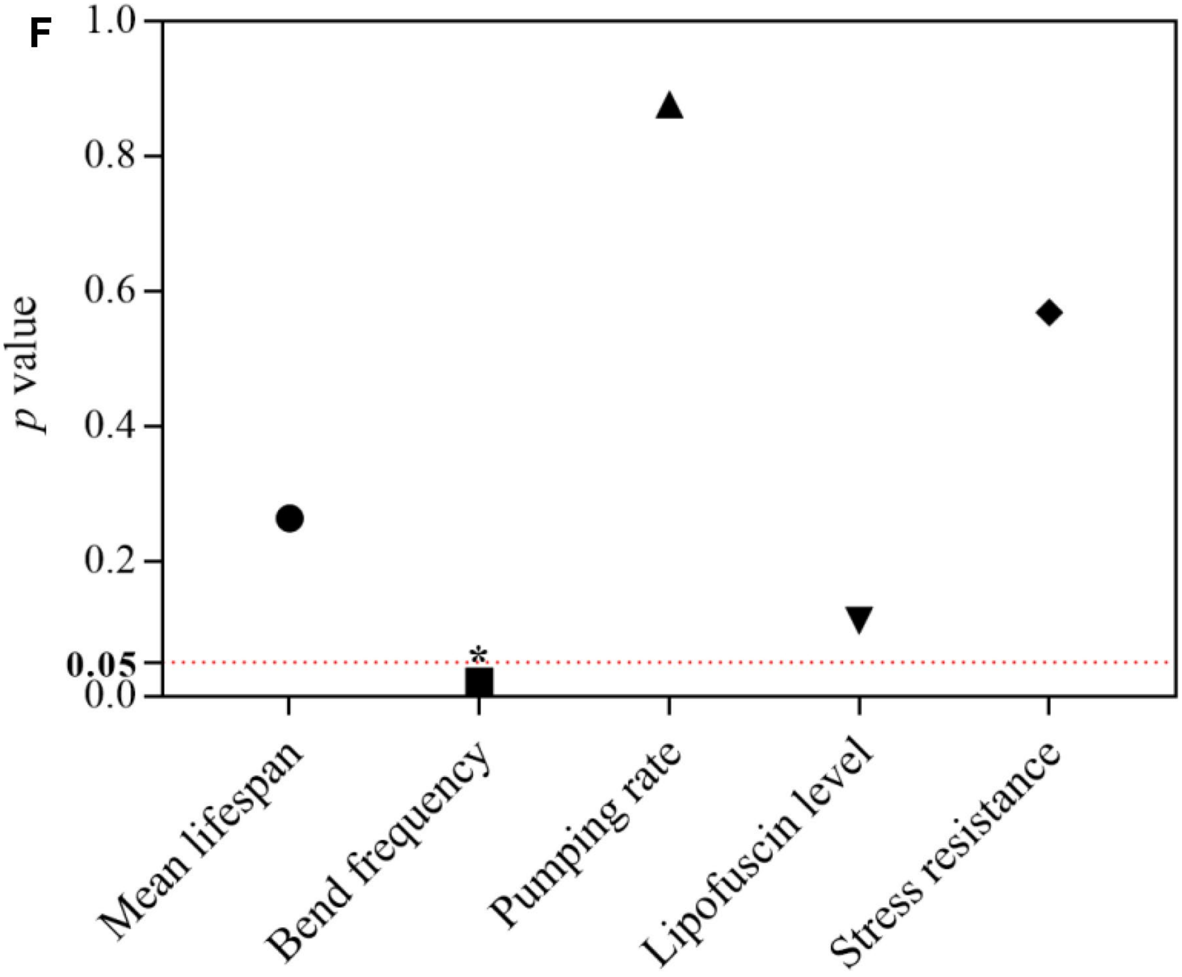

Supplementary Figure 2. Publication bias assessments. (**A**–**E**) showed the publication bias assessments of mean lifespan, bend frequency, pumping rate, lipofuscin level and stress resistance, respectively, while (**F**) summarized the risk of publication bias of each indicator. The sub-graph (I) was the funnel plots that directly exhibited the risk of publication bias. The sub-group (II) was the Egger's test expressed by line form that could quantitatively evaluate the risk of publication bias. Statistically significant publication bias was considered when the value of *p* was less than 0.05. *represented the appearance of significant publication bias ($p < 0.05$).

SUPPLEMENTARY REFERENCES

1. Akhoon BA, Pandey S, Tiwari S, Pandey R. Withanolide A offers neuroprotection, ameliorates stress resistance and prolongs the life expectancy of *Caenorhabditis elegans.* Exp Gerontol. 2016; 78:47–56. https://doi.org/10.1016/j.exger.2016.03.004 PMID:26956478
2. Chou WY, Lin YC, Lee YH. Short-term starvation stress at young adult stages enhances meiotic activity of germ cells to maintain spermatogenesis in aged male *Caenorhabditis elegans.* Aging Cell. 2019; 18:e12930. https://doi.org/10.1111/acel.12930 PMID:30816005
3. Kim J, Shirasawa T, Miyamoto Y. The effect of TAT conjugated platinum nanoparticles on lifespan in a nematode *Caenorhabditis elegans model.* Biomaterials. 2010; 31:5849–54. https://doi.org/10.1016/j.biomaterials.2010.03.077 PMID:20434216
4. Kishimoto S, Uno M, Okabe E, Nono M, Nishida E. Environmental stresses induce transgenerationally inheritable survival advantages via germline-to-soma communication in *Caenorhabditis elegans.* Nat Commun. 2017; 8:14031. https://doi.org/10.1038/ncomms14031 PMID:28067237
5. Kogure A, Uno M, Ikeda T, Nishida E. The microRNA machinery regulates fasting-induced changes in gene expression and longevity in *Caenorhabditis elegans.* J Biol Chem. 2017; 292:11300–09. https://doi.org/10.1074/jbc.M116.765065 PMID:28507100
6. Kronberg MF, Clavijo A, Moya A, Rossen A, Calvo D, Pagano E, Munarriz E. Glyphosate-based herbicides modulate oxidative stress response in the nematode *Caenorhabditis elegans.* Comp Biochem Physiol C Toxicol Pharmacol. 2018; 214:1–8. https://doi.org/10.1016/j.cbpc.2018.08.002 PMID:30142450
7. Olsen A, Vantipalli MC, Lithgow GJ. Lifespan extension of *Caenorhabditis elegans* following repeated mild hormetic heat treatments. Biogerontology. 2006; 7:221–30. https://doi.org/10.1007/s10522-006-9018-x PMID:16826446
8. Pandey S, Tiwari S, Kumar A, Niranjan A, Chand J, Lehri A, Chauhan PS. Antioxidant and anti-aging potential of Juniper berry (*Juniperus communis* L.) essential oil in *Caenorhabditis elegans* model system. Ind Crops Prod. 2018; 120:113–22. https://doi.org/10.1016/j.indcrop.2018.04.066
9. Pietsch K, Saul N, Chakrabarti S, Stürzenbaum SR, Menzel R, Steinberg CE. Hormetins, antioxidants and prooxidants: defining quercetin-, caffeic acid- and rosmarinic acid-mediated life extension in *C. elegans.* Biogerontology. 2011; 12:329–47. https://doi.org/10.1007/s10522-011-9334-7 PMID:21503726
10. Rathor L, Akhoon BA, Pandey S, Srivastava S, Pandey R. Folic acid supplementation at lower doses increases oxidative stress resistance and longevity in *Caenorhabditis elegans.* Age (Dordr). 2015; 37:113. https://doi.org/10.1007/s11357-015-9850-5 PMID:26546011
11. Rathor L, Pandey R. Age-induced diminution of free radicals by Boeravinone B in *Caenorhabditis elegans.* Exp Gerontol. 2018; 111:94–106. https://doi.org/10.1016/j.exger.2018.07.005 PMID:30004006
12. Schmeisser S, Schmeisser K, Weimer S, Groth M, Priebe S, Fazius E, Kuhlow D, Pick D, Einax JW, Guthke R, Platzer M, Zarse K, Ristow M. Mitochondrial hormesis links low-dose arsenite exposure to lifespan extension. Aging Cell. 2013; 12:508–17. https://doi.org/10.1111/acel.12076 PMID:23534459
13. Govindan S, Amirthalingam M, Duraisamy K, Govindhan T, Sundararaj N, Palanisamy S. Phytochemicals-induced hormesis protects *Caenorhabditis elegans* against α-synuclein protein aggregation and stress through modulating HSF-1 and SKN-1/Nrf2 signaling pathways. Biomed Pharmacother. 2018; 102:812–22. https://doi.org/10.1016/j.biopha.2018.03.128 PMID:29605769
14. Shukla V, Yadav D, Phulara SC, Gupta MM, Saikia SK, Pandey R. Longevity-promoting effects of 4-hydroxy-E-globularinin in *Caenorhabditis elegans.* Free Radic Biol Med. 2012; 53:1848–56. https://doi.org/10.1016/j.freeradbiomed.2012.08.594 PMID:23000058
15. Urban N, Tsitsipatis D, Hausig F, Kreuzer K, Erler K, Stein V, Ristow M, Steinbrenner H, Klotz LO. Non-linear impact of glutathione depletion on *C. elegans* life span and stress resistance. Redox Biol. 2017; 11:502–15. https://doi.org/10.1016/j.redox.2016.12.003 PMID:28086197
16. Wang D, Liu P, Xing X. Pre-treatment with mild UV irradiation increases the resistance of nematode *Caenorhabditis elegans* to toxicity on locomotion behaviors from metal exposure. Environ Toxicol Pharmacol. 2010a; 29:213–22. https://doi.org/10.1016/j.etap.2010.01.002 PMID:21787605

17. Wang X, Wang X, Li L, Wang D. Lifespan extension in *Caenorhabditis elegans* by DMSO is dependent on *sir-2.1* and *daf-16.* Biochem Biophys Res Commun. 2010b; 400:613–18. https://doi.org/10.1016/j.bbrc.2010.08.113 PMID:20828537

18. Wang D, Xing X. Pre-treatment with mild metal exposure suppresses the neurotoxicity on locomotion behavior induced by the subsequent severe metal exposure in *Caenorhabditis elegans.* Environ Toxicol Pharmacol. 2009; 28:459–64. https://doi.org/10.1016/j.etap.2009.07.008 PMID:21784043

19. Wu ZQ, Li K, Ma JK, Li ZJ. Effects of ethanol intake on anti-oxidant responses and the lifespan of *Caenorhabditis elegans.* CYTA J Food. 2019; 17:288–96. https://doi.org/10.1080/19476337.2018.1564794

20. Xu J, Guo Y, Sui T, Wang Q, Zhang Y, Zhang R, Wang M, Guan S, Wang L. Molecular mechanisms of anti-oxidant and anti-aging effects induced by convallatoxin in *Caenorhabditis elegans.* Free Radic Res. 2017; 51:529–44. https://doi.org/10.1080/10715762.2017.1331037 PMID:28503972

21. Yanase S, Ishii N. Hyperoxia exposure induced hormesis decreases mitochondrial superoxide radical levels via Ins/IGF-1 signaling pathway in a long-lived age-1 mutant of *Caenorhabditis elegans.* J Radiat Res (Tokyo). 2008; 49:211–18. https://doi.org/10.1269/jrr.07043 PMID:18285659

22. Yu X, Zhao W, Ma J, Fu X, Zhao ZJ. Beneficial and harmful effects of alcohol exposure on *Caenorhabditis elegans* worms. Biochem Biophys Res Commun. 2011; 412:757–62. https://doi.org/10.1016/j.bbrc.2011.08.053 PMID:21871869

23. Zhang J, Lu L, Zhou L. Oleanolic acid activates *daf-16* to increase lifespan in *Caenorhabditis elegans.* Biochem Biophys Res Commun. 2015; 468:843–49. https://doi.org/10.1016/j.bbrc.2015.11.042 PMID:26592451

24. Zhao X, Lu L, Qi Y, Li M, Zhou L. Emodin extends lifespan of *Caenorhabditis elegans* through insulin/IGF-1 signaling pathway depending on DAF-16 and SIR-2.1. Biosci Biotechnol Biochem. 2017; 81:1908–16. https://doi.org/10.1080/09168451.2017.1365592 PMID:28831863

25. Zhou L, Fu X, Jiang L, Wang L, Bai S, Jiao Y, Xing S, Li W, Ma J. Arbutin increases *Caenorhabditis elegans* longevity and stress resistance. PeerJ. 2017; 5:e4170. https://doi.org/10.7717/peerj.4170 PMID:29340230

26. Zhu CJ, Peng Y, Tong ZH, Lu LY, Cui YH, Yu HQ. Hormetic effect and mechanism of imidazolium-based ionic liquids on the nematode *Caenorhabditis elegans.* Chemosphere. 2016; 157:65–70. https://doi.org/10.1016/j.chemosphere.2016.05.007 PMID:27209554
