# Supplementary material for: Meta-analytic evidence for the anti-aging effect of hormesis on Caenorhabditis elegans
Source: Aging (Albany NY). 2020 Feb 7;12(3):2723–46. doi: 10.18632/aging.102773 (PMC7041774; doi:10.18632/aging.102773)
Supplement: Supplementary Table 1 [file aging-12-102773-s001..pdf]

SUPPLEMENTARY TABLE WITH REFERENCES

Supplementary Table 1. The basic information of included papers.

Reference	Treatment	Dose	Indicator	Source	Note
Akhoon et al., 2016 [1]	WA	5 µM	ML	Table 1	
			Lipofuscin	Fig. 1B	
			Bend	Fig. 2A	Day 1 adulthood
			Bend	Fig. 2A	Day 3 adulthood
			Bend	Fig. 2A	Day 5 adulthood
			daf-16	Fig. 9	
			sod-3	Fig. 9	
			hsp-16.2	Fig. 9	
Chou et al., 2019 [2]	STS	2-day	ML	Fig. S1B	Young-age
			ML	Fig. S1B	Mid-age
			ML	Fig. S1B	Repetitive fasting
			CML	Fig. S1B	Young-age
			CML	Fig. S1B	Mid-age
			CML	Fig. S1B	Repetitive fasting
			sod-3	Fig. 1e	Young-age
			sod-3	Fig. 1e	Mid-age
			sod-3	Fig. 1e	Repetitive fasting
Kim et al., 2010 [3]	[TAT-PtBP-Pt]	5 µM	Lipofuscin	Fig. 1L	
			ML	Table 2	
			OML	Table 2	
Kishimoto et al., 2017 [4]	As	1 mM	OML	Table S1	P0
			Bend	Fig. 2a	P0, day 4 adulthood
			Bend	Fig. 2a	P0, day 6 adulthood
	NaCl	150 mM	OML	Table S1	P0
			Bend	Fig. 2a	P0, day 4 adulthood
			Bend	Fig. 2a	P0, day 6 adulthood
	Fasting	1 day	OML	Table S1	P0

			Bend	Fig. 2a	P0, day 4 adulthood
			Bend	Fig. 2a	P0, day 6 adulthood
Kogure et al., 2017 [5]	IF	2-3 days	ML	Table 1	
			sod-3	Fig. 5	
Kronberg et al., 2018 [6]	Glyphosate F	4.8 mM	*sod-3*	Fig. 2	
			ctl-1	Fig. 2	
			ctl-1	Fig. 3A	
Olsen et al., 2006 [7]	RHS	33°C for 4 h	ML	Table 1	Treatment on the 4th and 8th day
			ML	Table 1	Treatment on the 4th, 8th and 12th day
			ML	Table 1	Treatment on the 4th, 8th, 12th and 16th day
			ML	Table 1	Treatment on the 8th and 12th day
			ML	Table 1	Treatment on the 8th, 12th and 16th day
			ML	Table 1	Treatment on the 4th and 12th day
			ML	Table 1	Treatment on the 4th and 16th day
			ML	Table 1	Treatment on the 12th and 16th day
Pandey et al., 2018 [8]	JBEO	10 ppm	ML	Table 2	
			Pumping	Fig. 4	Day 2 adulthood
			Pumping	Fig. 4	Day 5 adulthood
			Pumping	Fig. 4	Day 10 adulthood
			Lipofuscin	Fig. 7C	
Pietsch et al., 2011 [9]	Q	200 µM	ML	Table S1	
			Pumping	Fig. 3a	Day 3 adulthood
			Pumping	Fig. 3a	Day 6 adulthood
			Pumping	Fig. 3a	Day 10 adulthood
			Lipofuscin	Fig. 5h	
	CA	300 µM	ML	Table S2	
			Pumping	Fig. 3a	Day 3 adulthood
			Pumping	Fig. 3a	Day 6 adulthood
			Pumping	Fig. 3a	Day 10 adulthood
			Lipofuscin	Fig. 5h	

	RA	200 µM	ML	Table S3	
			Pumping	Fig. 3a	Day 3 adulthood
			Pumping	Fig. 3a	Day 6 adulthood
			Pumping	Fig. 3a	Day 10 adulthood
			Lipofuscin	Fig. 5h	
Rathor et al., 2015 [10]	FA	25 µM	ML	Table 1	
			Pumping	Fig. 2b	2-day-old
			Pumping	Fig. 2b	5-day-old
			Pumping	Fig. 2b	10-day-old
			Lipofuscin	Fig. 5	
			daf-16	Fig. 9	
			sod-3	Fig. 9	
Rathor and Pandey, 2018 [11]	BOB	25 µM	ML	Table 1	
			Pumping	Fig. 3A	Day 5 adulthood
			Pumping	Fig. 3A	Day 10 adulthood
			Bend	Fig. 3C	Day 5 adulthood
			Bend	Fig. 3C	Day 10 adulthood
			Lipofuscin	Fig. 7C	
			daf-16	Fig. 11	
			sod-3	Fig. 11	
			hsp-16.2	Fig. 11	
			ctl-1	Fig. 11	
Schmeisser et al., 2013 [12]	AsO_2	0.1 µM	ML	Table 1	
Shanmugam et al., 2018 [13]	Phytochemicals	300 µg/mL	ML	Table 1	
Shukla et al., 2012 [14]	4-HEG	20 µM	ML	Table 2	
			Pumping	Fig. 5A	Young
			Pumping	Fig. 5A	Day 5 adulthood
			hsp-16.2	Fig. 7	
			sod-3	Fig. 7	
Urban et al., 2017 [15]	DEM	100 µM	ML	Table 2	

			OML	Table 3	
			sod-3	Fig. 5A	Treatment for 1 day
			sod-3	Fig. 5A	Treatment for 2 days
			sod-3	Fig. 5A	Treatment for 3 days
			sod-3	Fig. 5A	Treatment for 5 days
			sod-3	Fig. 5A	Treatment for 7 days
			sod-3	Fig. 5A	Treatment for 10 days
			ctl-1	Fig. 5B	Treatment for 1 day
			ctl-1	Fig. 5B	Treatment for 2 days
			ctl-1	Fig. 5B	Treatment for 3 days
			ctl-1	Fig. 5B	Treatment for 5 days
			ctl-1	Fig. 5B	Treatment for 7 days
			ctl-1	Fig. 5B	Treatment for 10 days
Wang et al., 2010a [16]	Pre-treatment with UV irradiation	$10 J/m^2/min$	Bend	Fig. 3	Post-treatment with 50 µM Hg
			Bend	Fig. 3	Post-treatment with 100 µM Hg
			Bend	Fig. 3	Post-treatment with 200 µM Hg
			Bend	Fig. 3	Post-treatment with 50 µM Pb
			Bend	Fig. 3	Post-treatment with 100 µM Pb
			Bend	Fig. 3	Post-treatment with 200 µM Pb
			Bend	Fig. 3	Post-treatment with 50 µM Cr
			Bend	Fig. 3	Post-treatment with 100 µM Cr
			Bend	Fig. 3	Post-treatment with 200 µM Cr
Wang et al., 2010b [17]	DMSO	0.5%	ML	Table 1	
			HML	Fig. 2B	The first trial
			hsp-16.2	Fig. 2D	
Wang and Xing, 2009 [18]	Pre-treatment with Pb	2.5 µM	Bend	Fig. 3	Post-treatment with 50 µM Pb
			Bend	Fig. 3	Post-treatment with 100 µM Pb
	Pre-treatment with Hg	2.5 µM	Bend	Fig. 3	Post-treatment with 50 µM Hg
			Bend	Fig. 3	Post-treatment with 100 µM Hg
	Pre-treatment with Cu	2.5 µM	Bend	Fig. 3	Post-treatment with 50 µM Cu

			Bend	Fig. 3	Post-treatment with 100 µM Cu
	Pre-treatment with Cr	2.5 µM	Bend	Fig. 3	Post-treatment with 50 µM Cr
			Bend	Fig. 3	Post-treatment with 100 µM Cr
Wu et al., 2019 [19]	Ethanol	2%	ML	Table 2	
			Bend	Fig. 2	
			Pumping	Fig. 2	
Xu et al., 2017 [20]	Convallatoxin	20 µM	Pumping	Fig. 2C	4-day-old
			Pumping	Fig. 2C	6-day-old
			Pumping	Fig. 2C	8-day-old
			Pumping	Fig. 2C	10-day-old
			Pumping	Fig. 2C	12-day-old
			Lipofuscin	Fig. 2F	
			daf-16	Fig. 7	
			sod-3	Fig. 7	
Yanase and Ishii, 2008 [21]	Hyperoxia	90% oxygen	ML	Table 1	
Yu et al., 2011 [22]	Ethanol	2%	Bend	Fig. 2A	Treatment for 3 days
			Bend	Fig. 2A	Treatment for 5 days
			Bend	Fig. 2A	Treatment for 7 days
			Bend	Fig. 2A	Treatment for 9 days
			Bend	Fig. 2A	Treatment for 11 days
			Bend	Fig. 2A	Treatment for 13 days
			Bend	Fig. 2A	Treatment for 15 days
			Pumping	Fig. 2B	Treatment for 4 days
			Pumping	Fig. 2B	Treatment for 5 days
			Pumping	Fig. 2B	Treatment for 6 days
			Pumping	Fig. 2B	Treatment for 7 days
Zhang et al., 2015 [23]	OA	300 µM	ML	Table 1	
			OML	Table 2	
			Pumping	Fig. 3A	Day 3 adulthood
			Pumping	Fig. 3A	Day 6 adulthood

			Pumping	Fig. 3A	Day 10 adulthood
			daf-16	Fig. 4C	
			sod-3	Fig. 4C	
			hsp-16.2	Fig. 4C	
			ctl-1	Fig. 4C	
Zhao et al., 2017 [24]	Emodin	150 µM	ML	Table 1	
			OML	Table 2	
			HML	Table 2	
			sod-3	Fig. 3E	
			hsp-16.2	Fig. 3E	
			ctl-1	Fig. 3E	
Zhou et al., 2017 [25]	Arbutin	5 mM	ML	Fig. 1	
			HML	Fig. 2A	
			OML	Fig. 2B	
			UML	Fig. 2C	
			daf-16	Fig. 6A	
			sod-3	Fig. 6B	
			hsp-16.2	Fig. 6C	
Zhu et al., 2016 [26]	[OMMIM]Br	0.01 mg/L	ML	Fig. 2	
			Lipofuscin	Fig. 4A	Treatment for 2 days
			Lipofuscin	Fig. 4A	Treatment for 5 days
			Lipofuscin	Fig. 4A	Treatment for 7 days
			Lipofuscin	Fig. 4A	Treatment for 9 days
			Lipofuscin	Fig. 4A	Treatment for 11 days
			daf-16	Fig. 5B	Treatment for 2 days
			daf-16	Fig. 5B	Treatment for 5 days
			daf-16	Fig. 5B	Treatment for 7 days
			daf-16	Fig. 5B	Treatment for 9 days
			daf-16	Fig. 5B	Treatment for 11 days
	[DMMIM]Br	0.01 mg/L	ML	Fig. 2	

			Lipofuscin	Fig. 4A	Treatment for 2 days
			Lipofuscin	Fig. 4A	Treatment for 5 days
			Lipofuscin	Fig. 4A	Treatment for 7 days
			Lipofuscin	Fig. 4A	Treatment for 9 days
			Lipofuscin	Fig. 4A	Treatment for 11 days
			daf-16	Fig. 5B	Treatment for 2 days
			daf-16	Fig. 5B	Treatment for 5 days
			daf-16	Fig. 5B	Treatment for 7 days
			daf-16	Fig. 5B	Treatment for 9 days
			daf-16	Fig. 5B	Treatment for 11 days
	[DoMMIM]Br	0.01 mg/L	ML	Fig. 2	
			Lipofuscin	Fig. 4A	Treatment for 2 days
			Lipofuscin	Fig. 4A	Treatment for 5 days
			Lipofuscin	Fig. 4A	Treatment for 7 days
			Lipofuscin	Fig. 4A	Treatment for 9 days
			Lipofuscin	Fig. 4A	Treatment for 11 days
			daf-16	Fig. 5B	Treatment for 2 days
			daf-16	Fig. 5B	Treatment for 5 days
			daf-16	Fig. 5B	Treatment for 7 days
			daf-16	Fig. 5B	Treatment for 9 days
			daf-16	Fig. 5B	Treatment for 11 days

Abbreviation: CML, mean lifespan measured under cold stress; HML, mean lifespan measured under heat stress; NML, mean lifespan measured under normal condition; UML, mean lifespan measured under UV irradiation stress; *ctl-1*, *catalase-1*; *daf-16*, *dauer formation protein-16*; *hsp-16.2*, *small heat shock protein-16.2*; *sod-3*, *superoxide dismutase-3*. As, Arsenic; BOB, Boeravinone B; CA, Caffeic acid; DEM, Diethyl maleate; [DMMIM]Br, 1-decyl-2-methyl-3-methyl-imidazolium bromine; DMSO, Dimethyl sulfoxide; [DoMMIM]Br, 1-dodecyl-2- methyl-3-methyl- imidazolium bromine; FA, Folic acid; IF, Intermittent fasting; JBEO, Juniper berry essential oil; OA, Oleanolic acid; [OMMIM]Br, 1-octyl-2-methyl-3-methyl-imidazolium bromine; Q, Quercetin; RA, Rosmarinic acid; RHS, Repeated heat shock; STS, Short-term starvation; [TAT-PtBP-Pt], TAT-conjugated nano-Pt; WA, Withanolide A; 4-HEG, 4-Hydroxy-E-globularinin.

SUPPLEMENTARY REFERENCES

1. Akhoon BA, Pandey S, Tiwari S, Pandey R. Withanolide A offers neuroprotection, ameliorates stress resistance and prolongs the life expectancy of *Caenorhabditis elegans.* Exp Gerontol. 2016; 78:47–56. https://doi.org/10.1016/j.exger.2016.03.004 PMID:26956478
2. Chou WY, Lin YC, Lee YH. Short-term starvation stress at young adult stages enhances meiotic activity of germ cells to maintain spermatogenesis in aged male *Caenorhabditis elegans.* Aging Cell. 2019; 18:e12930. https://doi.org/10.1111/acel.12930 PMID:30816005
3. Kim J, Shirasawa T, Miyamoto Y. The effect of TAT conjugated platinum nanoparticles on lifespan in a nematode *Caenorhabditis elegans model.* Biomaterials. 2010; 31:5849–54. https://doi.org/10.1016/j.biomaterials.2010.03.077 PMID:20434216
4. Kishimoto S, Uno M, Okabe E, Nono M, Nishida E. Environmental stresses induce transgenerationally inheritable survival advantages via germline-to-soma communication in *Caenorhabditis elegans.* Nat Commun. 2017; 8:14031. https://doi.org/10.1038/ncomms14031 PMID:28067237
5. Kogure A, Uno M, Ikeda T, Nishida E. The microRNA machinery regulates fasting-induced changes in gene expression and longevity in *Caenorhabditis elegans.* J Biol Chem. 2017; 292:11300–09. https://doi.org/10.1074/jbc.M116.765065 PMID:28507100
6. Kronberg MF, Clavijo A, Moya A, Rossen A, Calvo D, Pagano E, Munarriz E. Glyphosate-based herbicides modulate oxidative stress response in the nematode *Caenorhabditis elegans.* Comp Biochem Physiol C Toxicol Pharmacol. 2018; 214:1–8. https://doi.org/10.1016/j.cbpc.2018.08.002 PMID:30142450
7. Olsen A, Vantipalli MC, Lithgow GJ. Lifespan extension of *Caenorhabditis elegans* following repeated mild hormetic heat treatments. Biogerontology. 2006; 7:221–30. https://doi.org/10.1007/s10522-006-9018-x PMID:16826446
8. Pandey S, Tiwari S, Kumar A, Niranjan A, Chand J, Lehri A, Chauhan PS. Antioxidant and anti-aging potential of Juniper berry (*Juniperus communis* L.) essential oil in *Caenorhabditis elegans* model system. Ind Crops Prod. 2018; 120:113–22. https://doi.org/10.1016/j.indcrop.2018.04.066
9. Pietsch K, Saul N, Chakrabarti S, Stürzenbaum SR, Menzel R, Steinberg CE. Hormetins, antioxidants and prooxidants: defining quercetin-, caffeic acid- and rosmarinic acid-mediated life extension in *C. elegans.* Biogerontology. 2011; 12:329–47. https://doi.org/10.1007/s10522-011-9334-7 PMID:21503726
10. Rathor L, Akhoon BA, Pandey S, Srivastava S, Pandey R. Folic acid supplementation at lower doses increases oxidative stress resistance and longevity in *Caenorhabditis elegans.* Age (Dordr). 2015; 37:113. https://doi.org/10.1007/s11357-015-9850-5 PMID:26546011
11. Rathor L, Pandey R. Age-induced diminution of free radicals by Boeravinone B in *Caenorhabditis elegans.* Exp Gerontol. 2018; 111:94–106. https://doi.org/10.1016/j.exger.2018.07.005 PMID:30004006
12. Schmeisser S, Schmeisser K, Weimer S, Groth M, Priebe S, Fazius E, Kuhlow D, Pick D, Einax JW, Guthke R, Platzer M, Zarse K, Ristow M. Mitochondrial hormesis links low-dose arsenite exposure to lifespan extension. Aging Cell. 2013; 12:508–17. https://doi.org/10.1111/acel.12076 PMID:23534459
13. Govindan S, Amirthalingam M, Duraisamy K, Govindhan T, Sundararaj N, Palanisamy S. Phytochemicals-induced hormesis protects *Caenorhabditis elegans* against α-synuclein protein aggregation and stress through modulating HSF-1 and SKN-1/Nrf2 signaling pathways. Biomed Pharmacother. 2018; 102:812–22. https://doi.org/10.1016/j.biopha.2018.03.128 PMID:29605769
14. Shukla V, Yadav D, Phulara SC, Gupta MM, Saikia SK, Pandey R. Longevity-promoting effects of 4-hydroxy-E-globularinin in *Caenorhabditis elegans.* Free Radic Biol Med. 2012; 53:1848–56. https://doi.org/10.1016/j.freeradbiomed.2012.08.594 PMID:23000058
15. Urban N, Tsitsipatis D, Hausig F, Kreuzer K, Erler K, Stein V, Ristow M, Steinbrenner H, Klotz LO. Non-linear impact of glutathione depletion on *C. elegans* life span and stress resistance. Redox Biol. 2017; 11:502–15. https://doi.org/10.1016/j.redox.2016.12.003 PMID:28086197
16. Wang D, Liu P, Xing X. Pre-treatment with mild UV irradiation increases the resistance of nematode *Caenorhabditis elegans* to toxicity on locomotion behaviors from metal exposure. Environ Toxicol Pharmacol. 2010a; 29:213–22. https://doi.org/10.1016/j.etap.2010.01.002 PMID:21787605

17. Wang X, Wang X, Li L, Wang D. Lifespan extension in *Caenorhabditis elegans* by DMSO is dependent on *sir-2.1* and *daf-16.* Biochem Biophys Res Commun. 2010b; 400:613–18. https://doi.org/10.1016/j.bbrc.2010.08.113 PMID:20828537

18. Wang D, Xing X. Pre-treatment with mild metal exposure suppresses the neurotoxicity on locomotion behavior induced by the subsequent severe metal exposure in *Caenorhabditis elegans.* Environ Toxicol Pharmacol. 2009; 28:459–64. https://doi.org/10.1016/j.etap.2009.07.008 PMID:21784043

19. Wu ZQ, Li K, Ma JK, Li ZJ. Effects of ethanol intake on anti-oxidant responses and the lifespan of *Caenorhabditis elegans.* CYTA J Food. 2019; 17:288–96. https://doi.org/10.1080/19476337.2018.1564794

20. Xu J, Guo Y, Sui T, Wang Q, Zhang Y, Zhang R, Wang M, Guan S, Wang L. Molecular mechanisms of anti-oxidant and anti-aging effects induced by convallatoxin in *Caenorhabditis elegans.* Free Radic Res. 2017; 51:529–44. https://doi.org/10.1080/10715762.2017.1331037 PMID:28503972

21. Yanase S, Ishii N. Hyperoxia exposure induced hormesis decreases mitochondrial superoxide radical levels via Ins/IGF-1 signaling pathway in a long-lived age-1 mutant of *Caenorhabditis elegans.* J Radiat Res (Tokyo). 2008; 49:211–18. https://doi.org/10.1269/jrr.07043 PMID:18285659

22. Yu X, Zhao W, Ma J, Fu X, Zhao ZJ. Beneficial and harmful effects of alcohol exposure on *Caenorhabditis elegans* worms. Biochem Biophys Res Commun. 2011; 412:757–62. https://doi.org/10.1016/j.bbrc.2011.08.053 PMID:21871869

23. Zhang J, Lu L, Zhou L. Oleanolic acid activates *daf-16* to increase lifespan in *Caenorhabditis elegans.* Biochem Biophys Res Commun. 2015; 468:843–49. https://doi.org/10.1016/j.bbrc.2015.11.042 PMID:26592451

24. Zhao X, Lu L, Qi Y, Li M, Zhou L. Emodin extends lifespan of *Caenorhabditis elegans* through insulin/IGF-1 signaling pathway depending on DAF-16 and SIR-2.1. Biosci Biotechnol Biochem. 2017; 81:1908–16. https://doi.org/10.1080/09168451.2017.1365592 PMID:28831863

25. Zhou L, Fu X, Jiang L, Wang L, Bai S, Jiao Y, Xing S, Li W, Ma J. Arbutin increases *Caenorhabditis elegans* longevity and stress resistance. PeerJ. 2017; 5:e4170. https://doi.org/10.7717/peerj.4170 PMID:29340230

26. Zhu CJ, Peng Y, Tong ZH, Lu LY, Cui YH, Yu HQ. Hormetic effect and mechanism of imidazolium-based ionic liquids on the nematode *Caenorhabditis elegans.* Chemosphere. 2016; 157:65–70. https://doi.org/10.1016/j.chemosphere.2016.05.007 PMID:27209554
